# Where did the herds go? Combining zooarchaeological and isotopic data to examine animal management in ancient Thessaly (Greece)

**DOI:** 10.1371/journal.pone.0299788

**Published:** 2024-10-22

**Authors:** Dimitris Filioglou, Silvia Valenzuela-Lamas, William P. Patterson, Leopoldo D. Pena, Samantha Presslee, Sandra Timsic, Antonio Delgado Huertas, Wietske Prummel, Canan Çakirlar

**Affiliations:** 1 Groningen Institute of Archaeology, University of Groningen, Groningen, The Netherlands; 2 Archaeology of Social Dynamics, Institució Milà i Fontanals d’Humanitats, Consejo Superior de Investigaciones Científicas, Barcelona, Spain; 3 Saskatchewan Isotope Lab, University of Saskatchewan, Saskatoon, Canada; 4 Departament de Dinàmica de la Terra i de l’Oceà, GRC Geociències Marines, Facultat de Ciències de la Terra, Universitat de Barcelona, Barcelona, Spain; 5 Department of Archaeology, BioArCh, University of York, Heslington York, United Kingdom; 6 Instituto Andaluz de Ciencias de la Tierra, Granada, Spain; University of Padova: Universita degli Studi di Padova, ITALY

## Abstract

Historians and archaeologists have been debating the scale of animal husbandry in ancient Greece for decades. This study contributes to the debate by examining Classical and Hellenistic faunal assemblages from Magoula Plataniotki, New Halos, and Pherae through non-destructive zooarchaeological methods and a multi-isotopic (^87^Sr/^86^Sr, δ^13^C, and δ^18^O) approach. Zooarchaeological data suggest that small-scale sedentary animal husbandry focused on caprine production in Magoula Plataniotiki and New Halos, and small-scale and semi-specialised animal husbandry was practised in Pherae. Isotopic data show both sedentary and mobile management of livestock in all sites, indicating different levels of production intensity and variety of goals. Based on our results, we propose an economic model whereby semi-specialised and small-scale animal husbandry co-existed, confirming mixed husbandry models for ancient Greece.

## Introduction

Researchers are divided over the scale of animal husbandry in Classical and Hellenistic Greece, as both historical and archaeological data are open to interpretation [1]. Some scholars argue for an “agropastoral model”, with households owning small, mixed herds [2, 3] that pastured close to settlements, manured the fields, and supplied daily needs [4, 5]. Animal and plant husbandry were practised side by side, wherein the absence of markets and a low demand for pastoral products (i.e. cheese/hair) [6] made full-time pastoralism and long-distance seasonal movements (transhumance) unnecessary and economically risky [1].

Other scholars, of the “transhumance model”, argue that increased urbanisation especially during the Hellenistic period raised the demand for meat for sacrifices, wool/fleece, and dairy products [7] making long-distance seasonal movements of large herds viable [8]. Animal and plant husbandry, they argue, were practiced by specialists [9] to produce surplus, supply the market, and generate profit [e.g. 10].

The archaeologists Reinders and Prummel first argued that New Halos herders practised seasonal movements (transhumance) between the Almiros and Sourpi Plains and the Othrys Mountains during the 3^rd^ century BCE [7]. They suggest that the increased urbanisation in the area, as seen through the archaeological evidence, increased demand for meat and animal byproducts. Environments suitable for transhumant movements could ensure enough pastureland for the large herds and maximise their productivity; similar practices were followed until recently in that region by mobile and semi-mobile pastoralists, while ancient sources imply pastoral activities and seasonal movements of flocks in the Othrys Mountains in antiquity. The authors interpret the predominance of evergreen vegetation over woodland [11, 12] and implications of dairy production [7, 13], as seen through the archaeobotanical and zooarchaeological record, as evidence of transhumance in the Hellenistic Period. Reinders and Prummel later reaffirmed their argument about transhumance between the Almiros and Sourpi Plains and the Othrys Mountains by presenting the archaeological and ethnographic evidence in more detail [14]. However, whether the change of vegetation is a result of extensive pasturing or timbering for construction (or both) is not yet clear. In addition, the zooarchaeological evidence is not sufficient to support an extensive use of caprines for dairy production.

Criticising these dichotomous models, others argue that both small-scale and large-scale specialised agropastoral activities co-existed in ancient Greece [15, 16], allowing to meet both the subsistence requirements of households in rural settlements and the demands of the elite (mostly living in larger centres) for profit and self-promotion [10, 17, 18]. Available pasture was sufficient to support both transhumance and sedentary animal husbandry practices [19].

For example, Bishop and colleagues utilised isotopic analyses and combined it with archaeological, textual, and ethnographic evidence to characterise animal management and mobility in the sites of Kastro Kallithea and Pharsalos in Hellenistic southern Thessaly [20, 21]. Their analysis demonstrated a diverse caprine management system that possibly targeted both subsistence and market consumption; some individuals were sedentary and raised locally for small-scale production, while others moved in different regions seasonally. Additionally, they identified transhumant animal management, partially confirming Reinders and Prummel’s suggestions that transhumant movements were practised in the wider area at that time. Nevertheless, this work is time- and site-restricted, as it exclusively concerns the animal management of two sites during the 3^rd^ and 2^nd^ centuries BCE. Furthermore, it overlooks the potential impacts of continuous warfare in the wider region on local economies.

**Fig 1:**
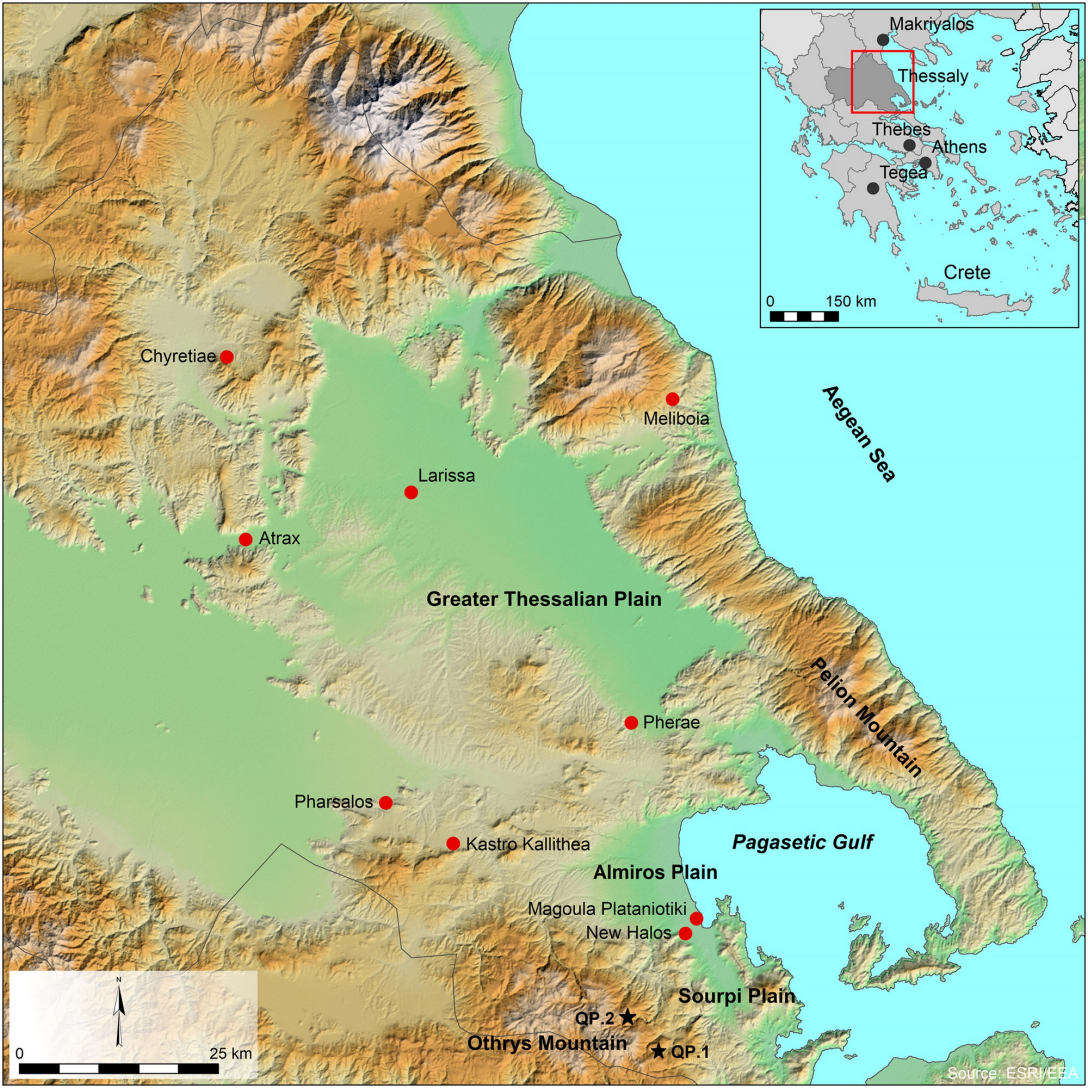
Sites mentioned in the text (red dots) and samples collected for Sr analysis (black stars). Map prepared by Erwin Bolhuis using ArcGIS 10.2 under a CC BY license, with permission from Erwin Bolhuis, original copyright 2023.

The current study takes part in this discussion with new zooarchaeological (taxonomic composition and age-at-death) and isotopic (strontium ^87^Sr/^86^Sr, carbon δ^13^C and oxygen δ^18^O) evidence from three settlement sites: Magoula Plataniotiki, New Halos, and Pherae (Fig 1) dating from the Classical through the Hellenistic periods (4^th^-1^st^ centuries BCE) in southern Thessaly. Although the dataset has its limitations (due to differential excavation methods, sample size, and arbitrary contexts), we infer the degree of herd mobility across this period in southern Thessaly and integrate its implication for the modes of animal husbandry strategies in the Classical and Hellenistic economy.

## The sites, their economies and the cultural landscape of Thessaly

Located in central Greece, Thessaly was an important passage between the north and south, as well as the west (mainland) and the east (island) Greece. Classical and Hellenistic authors already recognised Thessaly’s agricultural wealth and consequent economic and political importance in ancient Greece [22]. Ancient texts mention Thessaly exporting grain to Thebes [23], Kos (*RFIC* 62, 1934, 169), Athens [24], and Rome [25]. It is not a coincidence that wheat was depicted on the Thessalian League’s coins in the 2^nd^ century CE [26]. The location and fertility of Thessaly caused extensive instability due to the rivalry among the local elites as well as different leagues and empires to control the resources and the strategic locations of this area [27].

The fertile lands of Thessaly provided rich pasture for sheep, cattle, and horses as well [28]. The Thessalian poet Theocritus mentions that Thessalian elite, which played a central role in the sociopolitical life of Thessaly, owned herds of thousands of sheep and cattle [29]. However, it is likely that written sources focus on elite practices and often exaggerate [21]; they should be approached with caution [30]. Moreover, the entirety of Thessaly was not rich in grain; the periphery (perioikoi) was covered by mountainous areas and infertile lands unsuitable for dry agriculture. Ancient sources associate perioikoi with pastoralism [31].

The towns of southeastern Thessaly offer a unique opportunity to examine the agropastoral models, as they neighbour with diverse landscapes (mountains, plains, the Pagasetic Gulf). Magoula Plataniotiki [e.g. 32, 33] and New Halos [14, 31, 34–36] were located between Almiros and Sourpi (Fig 1), two small plains of moderate fertility [37] in Achaia Phthiotis, one of the perioikoi in southeast Thessaly [27]. A few kilometres north of Almiros, Pherae was located at the edge of the Greater Thessalian Plain.

The modest town of Magoula Plataniotiki was built on a beach ridge [35] next to a salty marsh and the Pagasetic Gulf and inhabited from at least the 6^th^ to the 2^nd^ centuries BCE. Its extent is estimated at 10 ha and it may have been inhabited by around 2000 people [38]. Around 346 BCE, the Macedonian army destroyed the town and gave it to the neighbouring town of Pharsalos. However, pottery and architectural remains indicates that the town was still inhabited for a few decades after its destruction. The lack of luxurious items and evidence for intensive farming on the Almiros Plain [39] hint at a subsistence economy. Apart from some monumental buildings whose function is yet to be defined [33], most buildings are of moderate size, which confirms the lack of luxury in Magoula Plataniotiki. Only a small part of the site has been excavated systematically so far by a collaboration of Dutch (Vladimir Stissi, Tamara Dijkstra) and Greek (Yiannis Lolos, Vaso Rondiri) archaeologists revealing parts of domestic buildings and spaces.

New Halos was the largest (40 ha) and most crowded town in Almiros, with an estimated 9000 inhabitants [35]. The systematic excavation of eight houses during the 1990’s and 2000’s by the archaeologist Reinder Reinders revealed that the site was inhabited for around 35 years in the first half of the 3^rd^ century BCE in the foothills of the Othrys Mountains [14]. The site was built quickly and strategically with central planning on a narrow passage that connects northern with southern Greece and was fortified; as such, it likely served a military function [31, 40]. After it was abandoned, its southeast gate was converted into a farmhouse which was used for at least half a century [38]. The archaeologist and researcher on domestic economy Margriet Haagsma [31] argued that mixed farming and extensive pastoralism co-existed, while the domestic economies relied on pastoralism, a limited cultivation of grain, horticulture, and possibly, the cultivation of olives. Income produced from small-scale industries such as coroplastry and weaving could be used to import grain from central Thessaly for subsistence [31].

The city of Pherae was located in close vicinity to the Pagasetic Gulf and both the Othrys and Pelion Mountains (Fig 1). Pherae underwent important fluctuations in significance, size, and connectivity between 3^rd^-1^st^ centuries BCE, due to the regional instability caused by Macedonian and Roman military activity [e.g. 41–43]. The economic and political power of Pherae started to decline in the mid-4^th^ century BCE, when King Philippos B’ of Macedonia conquered the city [44]. At the beginning of the 2^nd^ century BCE, the Romans defeated the Macedonians, re-founded the Thessalian League (Thessaliko Koino), and designated the Pheraean general Pausanias Ehekratous as its leader marking a final period of prosperity [42, 45]. Pherae was gradually abandoned when the Romans incorporated the whole Thessaly into the Achaean province. Rescue excavations in the 1980’s and 2000’s by the local Ephorate of Antiquities and historical sources indicate that Pherae was a city involved in small industries (e.g. glass, pottery, copper objects, textile, figure, tool and glass production) and short- and long- distance trade (e.g. with Rome) in the Hellenistic Period [42, 45–53].

The initial zooarchaeological analysis in Magoula Plataniotiki and New Halos indicated small-scale, sedentary, unspecialised animal husbandry for subsistence in Magoula Plataniotiki and New Halos with little variation between periods and sites [54]; slightly different patterns were observed in Pherae, where sedentary, medium-scale, semi-specialised caprine rearing prevailed in the animal economy [55]. No Roman markers in animal husbandry (e.g. more intensive exploitation of cattle or size increase of animals) were identified in Pherae, despite the increasing involvement of the Romans in Thessalian politics from the late 3^rd^ century BCE onwards.

## Materials and methods

### Samples

This paper focuses on the faunal remains recovered by hand (i.e. no systematic sieving was employed in the assemblages). Assemblages come from:

a) four systematically excavated medium-size Hellenistic houses, other Classical domestic structures and “common areas” from Magoula Plataniotiki (4^th^-2^nd^ centuries BCE),
b) eight systematically excavated medium-size Hellenistic houses in New Halos (ca first half of the 3^rd^ centuries BCE), and
c) three small- to medium-size Late Hellenistic houses (V. Chadjitheodorou, E. Chadjitheodorou, and E. Tsoumbekou plots) and one relatively larger Hellenistic house with residential and workshop areas in Pherae (3^rd^ century BCE- 1^st^ century BCE), all revealed during rescue excavations.

To examine animal husbandry practices as a whole and because the sample sizes are not sufficiently large to permit quantitatively meaningful contextual analysis, we merge and analyse zooarchaeological data of all contexts in each site. The faunal materials of Magoula Plataniotiki and Pherae were analysed by Dimitris Filioglou following the protocol described in Filioglou, Prummel and Çakirlar [54] and Filioglou and Çakirlar [55] using a small reference collected at the dig house and museum respectively. We added the faunal remains recovered from the Classical Magoula Plataniotiki structures in Trench 6 during the 2021 campaign, excluding unstratified contexts. Wietske Prummel had analyzed the faunal remains from the eight houses of New Halos using a different protocol [13], her data were re-quantified for consistency.

### Macroscopic analysis

We combined the relative taxonomic frequency in Number of Identified Specimens (NISP) [56] with ageing data to identify the type and scale of production and trace the degree of animal mobility. A faunal assemblage with mixed taxonomic frequency would imply small-scale herding, whereas an assemblage dominated by one species would imply the reverse [57]. The intensive slaughtering of caprines and cattle around two years of age could indicate meat production; the slaughtering of very young individuals (up to two months old), together with high representation of older females, could indicate dairy production; keeping males alive for longer periods would reflect hair production. Moreover, the absence of stock from a site for a whole season could be identified by the distinct underrepresentation of specific age groups [58]. Finally, the presence of individuals that died perinatally might be indicative of animal penning [59]. As a proxy for investigating age-at-death of caprines we used mandibles with teeth embedded and loose lower teeth (dP4, P4-M3) after Payne [60, 61]. Due to the limited number of ageable pig and cattle teeth, we estimated age-at-death based on the fusion stages of post-cranial bones after Zeder, Lemoine and Payne [62] for pigs and after Reitz and Wing [63] for cattle.

As the separation of sheep and goat elements based on tooth morphology was not possible in some samples suitable for isotopic analysis [64], but important for interpreting animal mobility, four caprine teeth and mandibular bones (two from New Halos and two from Pherae) were analysed by Zooarchaeology by Mass Spectrometry (ZooMS) analysis to identify the species based on collagen-peptide sequencing [65–67]. The samples for ZooMS analysis were prepared and analysed following Presslee et al [68] at the BioArCh laboratory in York, UK (see S1 Text for a brief outline of the process).

### Isotope analysis

We conducted stable oxygen (δ^18^O), carbon (δ^13^C) and strontium (^87^Sr/^86^Sr) isotope analysis on the enamel bioapatite of animals to infer mobility, diet, and seasonality of movements respectively. Intra-individual amplitudes (Δ) of isotope ratios were compared to “normal” seasonality δ^13^C and δ^18^O values as well as ^87^Sr/^86^Sr variability indicative of different herding regimes (mobility/sedentary).

#### Stable carbon (δ^13^C) and oxygen (δ^18^Ο) isotopes

Together, δ^18^Ο and δ^13^C values of tooth enamel bioapatite provide valuable data for assessing livestock seasonal mobility [69, 70], diet [71, 72] and food resources [73]. C_3_ plants have δ^13^C values ranging between -36‰ and -24‰, whereas C_4_ plants have δ^13^C values ranging between -19‰ and -6‰ [74–76]. Due to tissue-specific fractionation, sheep and cattle enamel carbonate δ^13^C values are generally 11.5–15.4‰ higher than plants consumed [73, 77, 78]. δ^13^C of -8/-9‰ and below would reflect consumption of C_3_ plants, while δ^13^C above -8/-9‰ would reflect consumption of C_4_ plants. Seasonal Δ^13^C is expected to range between 1‰ and 3‰ [79, 80]; a combined C_3_ and C_4_ plant species is expected to display elevated δ^13^C values and thus higher Δ^13^C [76]. Although C_3_ species dominate wild Greek flora [81, 82], C_4_ species could be present as well; millet (*Panicum miliaceum*) has been found in antiquity in the wider region [83], while the coastal and marshy environments of the Almiros Plain and the Pagasetic Gulf could host halophytes (e.g. *Salsola kali*, *Crithmum maritimum*).

The δ^18^Ο values of local water pass to an animal body via ingested plant food and water [84, 85]. Geographic and climatic factors affect the δ^18^Ο value of meteoric water and thus the δ^18^Ο ratios locked into tooth enamel, with a known fractionation [e.g. 86–90]. In our study area, δ^18^Ο values at high latitudes increase during the drier/hotter (May-October) season and decrease during wetter/cooler (November-April) seasons [91, 92]. This relationship results because meteoric water at higher altitudes and inland/drier areas has lower δ^18^O values relative to meteoric water in lower altitudes and coastal/moister areas [93, 94]. Sequential analyses of enamel carbonate reflect the seasonal cycle in δ^18^O values during the period of tooth enamel formation [95].

Estimating the local “normal” seasonal amplitude of δ^18^O in an individual is challenging as the modern Δ^18^O in precipitation or expected meteoric water is much higher than the Δ^18^O of enamel carbonate [94]; as such, instead of establishing a standard range we grouped animals with similar Δ^18^O together to examine seasonal variation. In Thessaly, enamel δ^18^O values are expected to be lowest around December and at the highest around June [96]; an animal with limited mobility that grazes in fixed pastures is therefore expected to show a larger range in δ^18^O values than a migratory animal that is translocated to different altitudes seasonally [97, 98].

#### Strontium isotopes (^87^Sr/^86^Sr)

^87^Sr is produced by the decay of radioactive ^87^Rb found in the bedrocks, with resultant ^87^Sr/^86^Sr ratios dependent upon the age and original ^87^Rb content of the bedrock. Consequently, younger rocks tend to have lower ^87^Sr/^86^Sr ratios than older and more felsic rock [99]. Animals obtain ^87^Sr/^86^Sr ratios from plants and drinking water that have incorporated them from the soil and underlying bedrock [100, 101] with no fractionation [102, 103]. Sequential tooth enamel ^87^Sr/^86^Sr ratios therefore reflect individual’s food sources, whether they changed during enamel mineralisation, and thus may also reflect mobility patterns [79, 104, 105].

The intra-individual ^87^Sr/^86^Sr range up to 0.0003 is considered as indicative of sedentary animals in Mediterranean contexts [20, 106, 107]. Higher intra-individual variation (Δ) in ^87^Sr/^86^Sr or differences in ^87^Sr/^86^Sr between the burial place and the tooth enamel would indicate mobility [108]. The geology of southern Thessaly is too diverse to pin the pathways of movements across the region [109–112]. In this context, animals with ^87^Sr/^86^Sr values that overlap with the strontium isotope baseline were not systematically considered as “local” and we acknowledge that they may have pastured in another region with the same ^87^Sr/^86^Sr.

#### Sampling for isotope analysis

We sampled 23 individuals in total (Table 1). The sample size is limited by the availability of suitable molars. Specimens were relative-dated based on their stratigraphy and associated pottery, while a single goat specimen from Magoula Plataniotiki was directly radiocarbon dated, because it came from a mixed context. After the analysis, HA5 was grouped together with HA9 as pre-4^th^ century BCE (see S2 Text and S1 Table).

**Table 1:** Sampled teeth per site and species.

Site	Species			
	Cattle	Sheep	Goat	Total
Pre-4^th^ century BCE Magoula Plataniotiki	1	0	1	**2**
Classical Magoula Plataniotiki	0	3	0	**3**
Hellenistic Magoula Plataniotiki	1	2	0	**3**
Hellenistic New Halos	0	3	1	**4**
Hellenistic Pherae	2	8	1	**11**
Total	4	16	3	**23**

**Fig 2:**
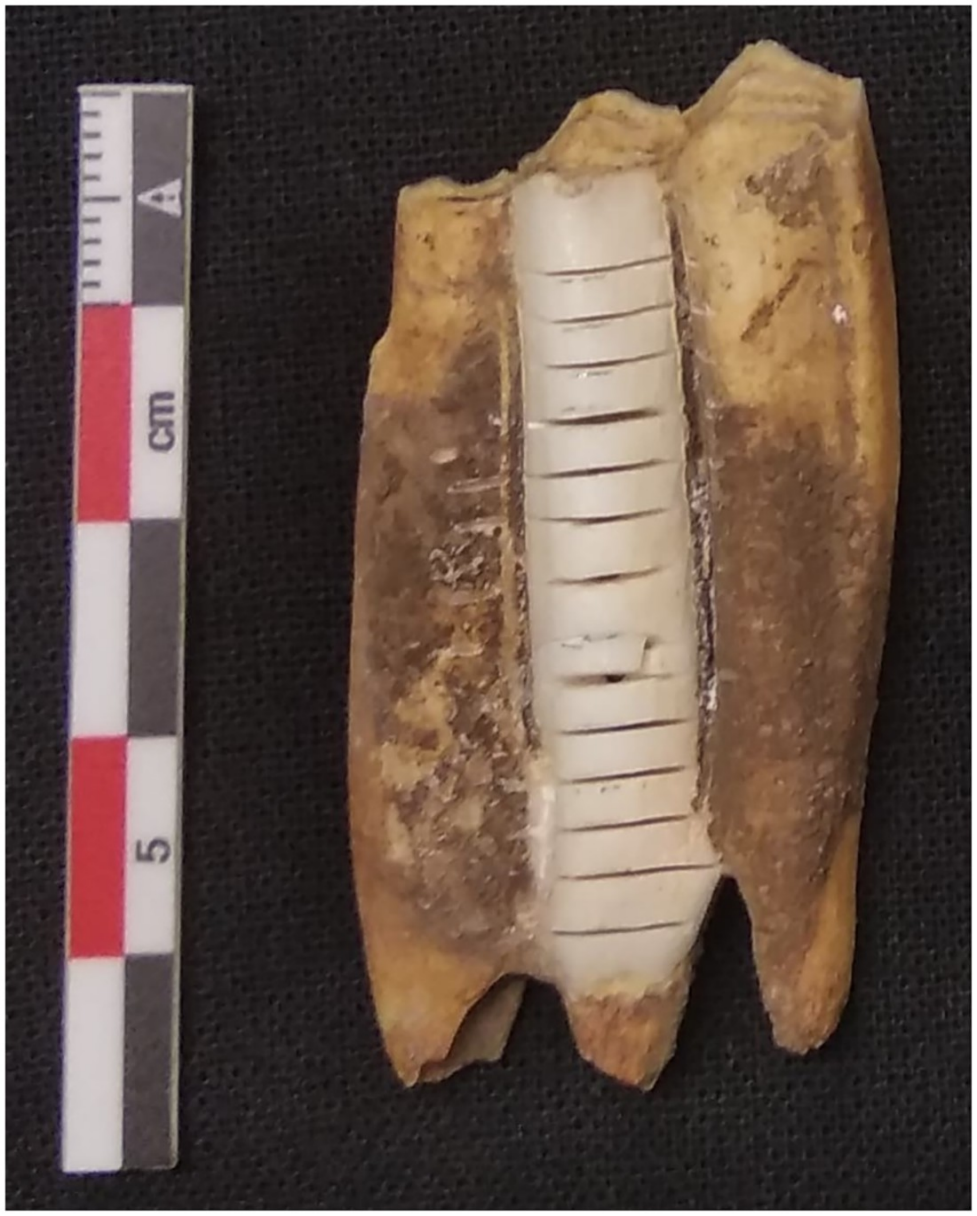
A sequentially sampled lower third molar. Photo by D. Filioglou.

We sequentially sampled the enamel of the middle pillar of lower third molars (Fig 2) to make our data comparable with previous studies on animal mobility [e.g. 113, 114]. When the middle pillar was broken, we sampled the anterior pillar of lower third molars or the posterior pillar of lower second molars. An unworn lower second molar (M2) of a caprine contains a 12-month record from the first to the second year of life and the lower third molar (M3) forms over a period of 20–24 months in the second and third years of an individual’s life [79, 115]. Crown formation of the second molar in cattle begins at approximately one month of age and is completed by one year of age, while the third molar begins between six months and one year of age and it is completed by two years of age on average [116, 117]. The number of samples per tooth ranged between 7 and 13 except HA9, which provided only four samples, because its crown was very short. Sampling strategy was designed to provide reliable results while preserving as much tooth material as possible for future studies [118].

The protocol used for sampling archaeological teeth follows Valenzuela et al [113]. We first mechanically removed cementum and sediment from the tooth enamel surface by abrasion using a tungsten carbide dental bur. Thereafter, transversal slices of enamel (~3mm wide each) were recovered from between 3mm above the enamel-root junction (ERJ) and below the occlusal surface using a diamond cutter disc. We also removed all of the dentine from the enamel using a dental drill. From each sample, we used a minimum of 6-7mg for δ^18^Ο and δ^13^C analyses and 12-13mg for ^87^Sr/^86^Sr analyses, with any remaining material archived for future research. Finally, we crushed the enamel slices using an agate mortar and weighed the enamel powder on a Kern ABP scale with an ionisation panel. An overview of the samples’ processing and isotopic measurement is briefly described in S3 and S4 Texts. All permission were issued by the Ephorate of Magnesia, Hellenic Ministry of Culture and Sports (S5 Text).

To establish a strontium isotope baseline and complement the strontium isotope maps of previous studies on the area [20], a total of seven samples were used. More specifically, one archaeological mandibular bone of sheep (HA2) and one of cattle (HA10) from Magoula Plataniotiki, one mandibular bone of sheep from New Halos (NH4), and one mandibular bone and dentine from two sheep (PH3 and PH11 respectively) from Pherae were analysed to set a baseline of local strontium values in each site as they are known to chemically equilibrate with the burial environment and thus reflect the strontium isotopic ratio of the soil [e.g. 100, 119, 120]. To assess the variability of bioavailable strontium ratios in the Othrys Mountains, four modern leaves (QP1.2, QP1.3, QP2.2, QP2.3) from four oak trees (*Quercus pubescens*) from two different geological formations were also analysed. We did not collect leaf samples from the plains around the sites due to current heavy anthropogenic activity. To avoid taking samples from the same geological layers as Bishop [20, 21], we sampled from younger geological substrates (Upper Cretaceous flysch and transgressive limestones). Isotopic data were processed using R language (R Core team) building from previous scripts [121].

Baseline leaf samples originated from forests on the peaks of the Othrys Mountains, far (>100m) from any human activity and streams or lakes, in the centre of a geological layer (see S1 Protocol in S1 File) for the detailed sampling protocol). The location of the sampled oak trees was recorded using a hand-held GPS device. It is expected that potential contribution of ^87^Sr/^86^Sr derived from Saharan dust plays a minor role in determining the bioavailable ^87^Sr/^86^Sr composition in Thessaly [122].

## Results

### Macroscopic analysis

**Fig 3:**
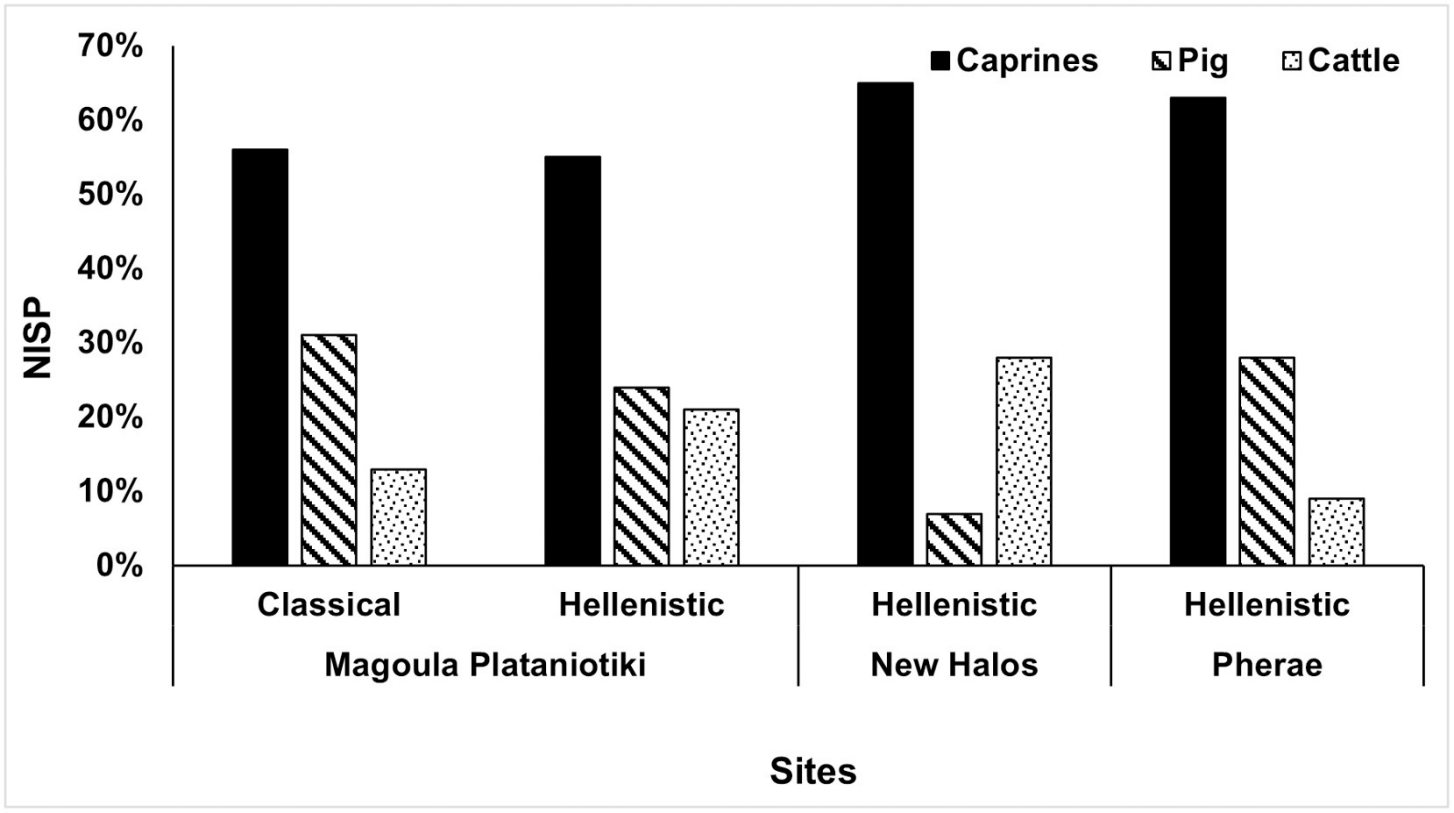
Relative taxonomic abundance per site and period (Classical Magoula Plataniotiki: n = 249,5 [ratio goat:sheep = 1:2.9]; Hellenistic Magoula Plataniotiki: n = 175,5 [ratio goat:sheep = 1:2.0]; New Halos: n = 150 [ratio goat:sheep = 1:3.3]; Pherae: n = 849 [ratio goat:sheep = 1.4.3]).

Caprine remains outnumber pig and cattle remains in NISP counts in all sites (Fig 3 and S2 Table). Pigs are the second most frequent species in Classical Magoula Plataniotiki and Pherae (ca 30%), while in New Halos they represent less than 10% of the main domesticates. Cattle remains, although infrequent in Classical Magoula Plataniotiki and Pherae, slightly increase in frequency in Hellenistic Magoula Plataniotiki and New Halos (ca 20%). Differences in the relative abundance of taxonomic groups between the Classical and Hellenistic Periods in Magoula Plataniotiki are statistically significant (chi-squared test: *p* = 0.04).

**Fig 4:**
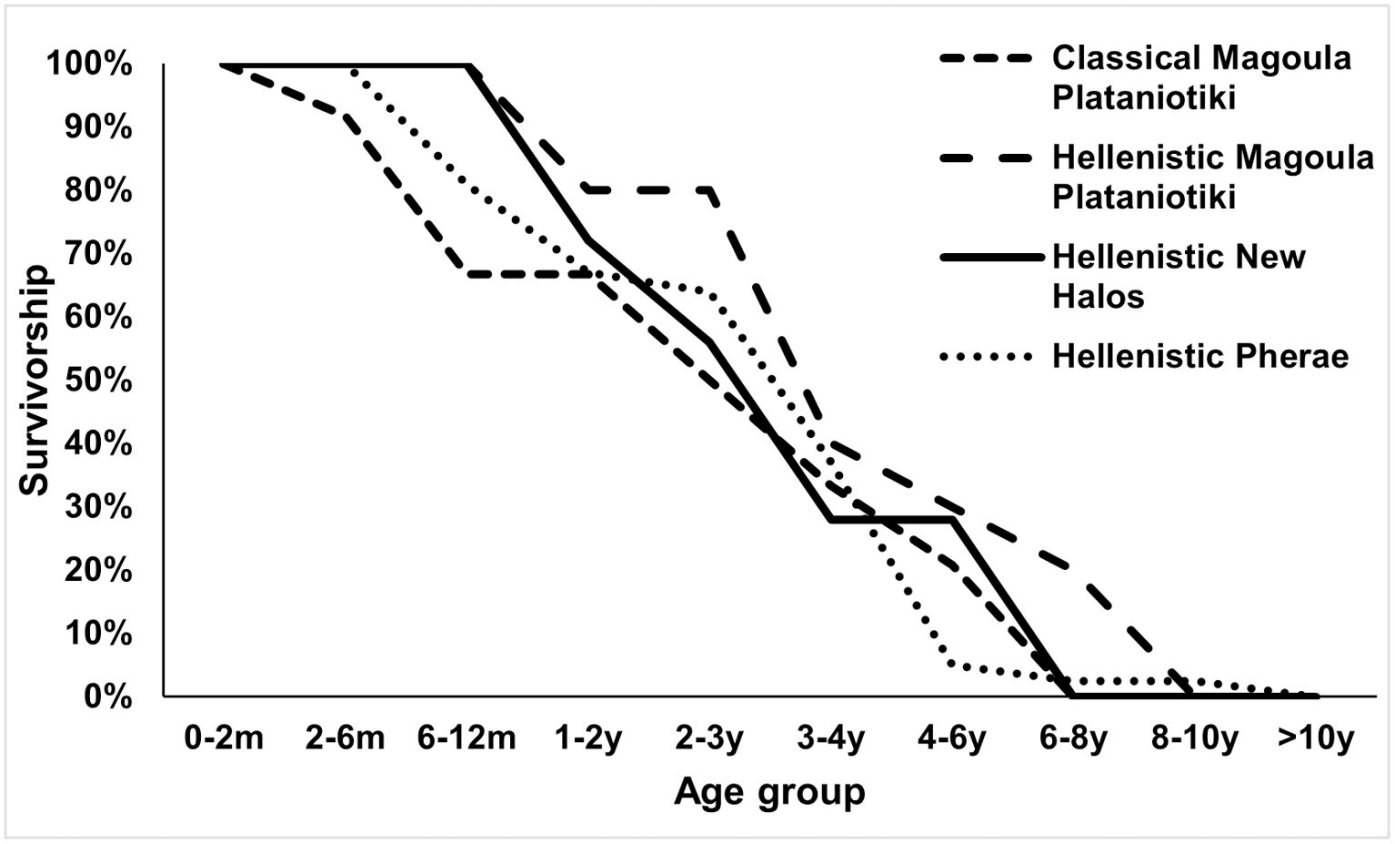
Survivorship curves of caprines based on tooth eruption and wear. Classical Magoula Plataniotiki: n = 12, Hellenistic Magoula Plataniotiki: n = 5, New Halos: n = 16; Pherae: n = 39.

Caprines were slaughtered at various stages of their lives in all sites (Fig 4 and S3 Table). Some individuals in Classical Magoula Plataniotiki (35%) were slaughtered before reaching their first year of age, a second group of individuals were slaughtered after their second year of age, with no individuals surviving beyond eight years. In Hellenistic Magoula Plataniotiki, the sample size is too small for sound patterns in age-at-death. All New Halos caprines were slaughtered after their first year of life; no individual survived beyond eight years. Regarding Pherae, one third of the caprine population was slaughtered between six months and two years of age. Almost 60% of the caprine population was slaughtered between the third and sixth years of age, while only 2% of the total population survived beyond six years.

**Fig 5:**
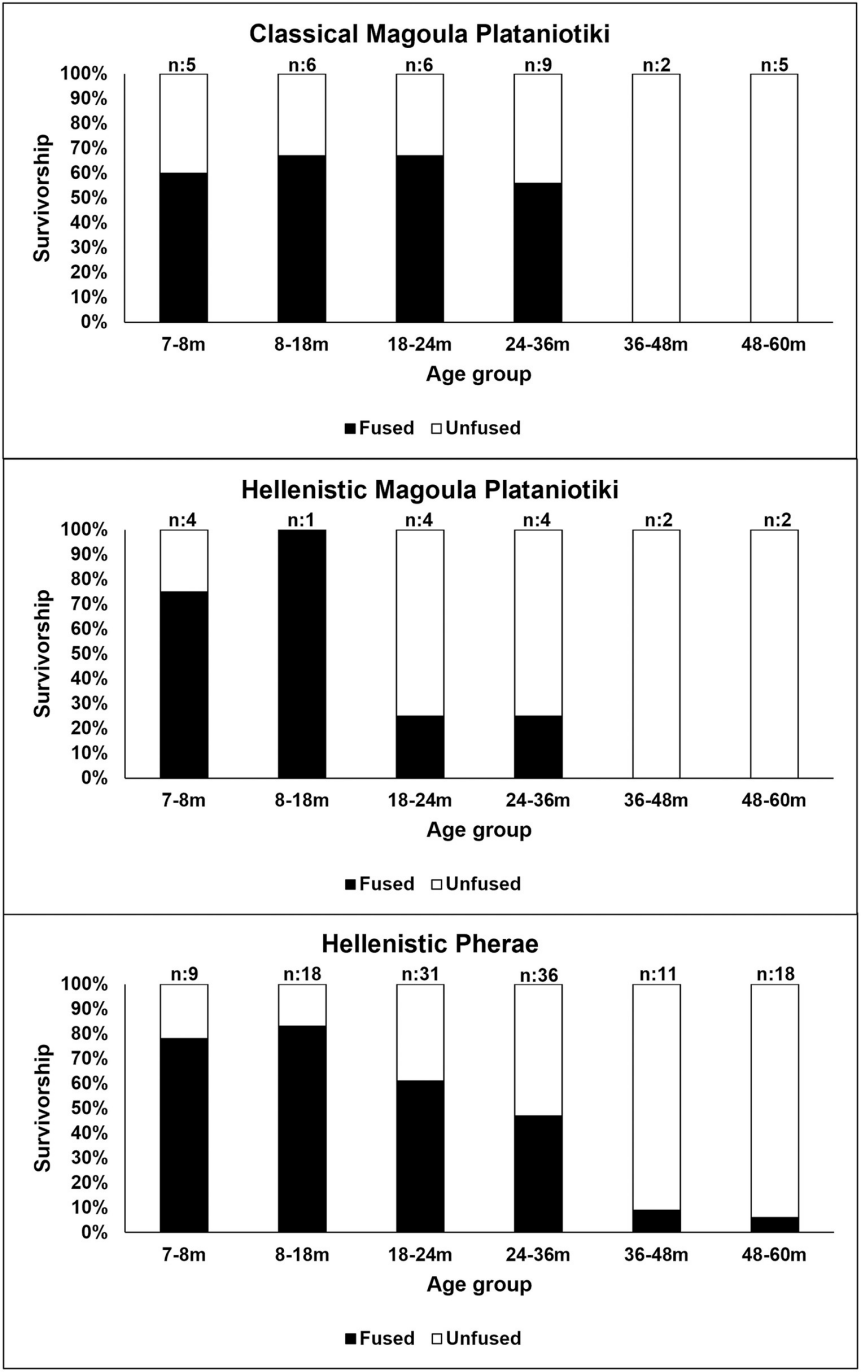
Mortality profiles of pigs based on epiphyseal fusion stage. Classical Magoula Plataniotiki: n = 33; Hellenistic Magoula Plataniotiki: n = 17; Pherae: n = 123.

**Fig 6:**
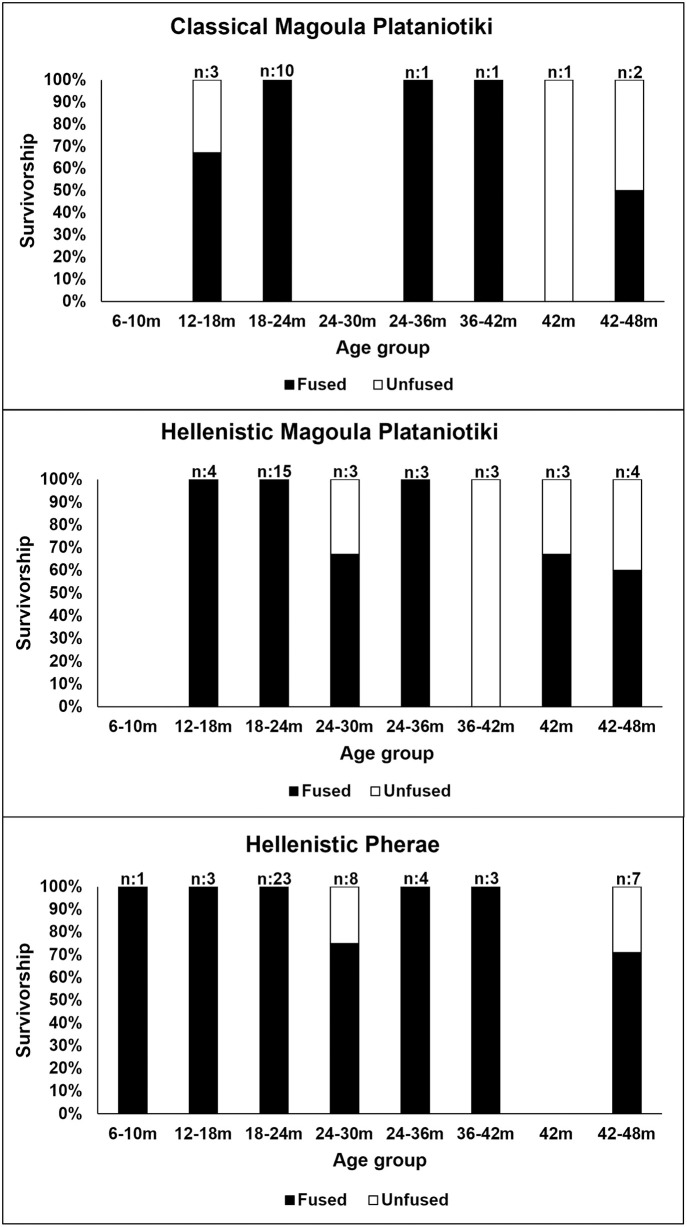
Mortality profiles of cattle based on epiphyseal fusion stage. Classical Magoula Plataniotiki: n = 18; Hellenistic Magoula Plataniotiki: n = 35; Pherae: n = 49.

Most pigs were slaughtered before three years of age in Magoula Plataniotiki and Pherae (Fig 5 and S4 Table), while fewer individuals were kept alive longer. However, the proportion of older individuals in Pherae is slightly higher compared to Magoula Plataniotiki. Pig mortality profiles from New Halos were omitted from the analysis due to the small sample size. The few available cattle remains from Magoula Plataniotiki produced incomplete mortality profiles (Fig 6 and S5 Table). The ageable cattle remains from New Halos were insufficient for analysis and thus omitted. Cattle in Magoula Plataniotiki and Pherae were slaughtered in different periods of their lives, while both sites had at least one unrepresented age group, probably due to the small sample size. It appears that cattle from Pherae were not killed frequently and were mostly kept alive until adulthood.

Finally, individuals who died perinatally or as infants were only represented by their post-cranial remains (Table 2). Very young caprines were most frequent in Classical Magoula Plataniotiki, while very young pigs comprised a quarter of the total pig remains in Hellenistic Magoula Plataniotiki. Very young cattle are only represented by one specimen in New Halos and Pherae.

**Table 2:** Frequency of perinatal and neonatal/ infant remains per site.

Species	Age-class	Classical Magoula Plataniotiki		Hellenistic Magoula Plataniotiki		New Halos		Pherae	
		n	%	n	%	n	%	n	%
**Caprines**	Perinatal	6	**5**	3	**3**	3	**3**	5	**1**
	Neonatal/infant	3	**3**	2	**2**	1	**1**	2	**0.5**
**Pigs**	Perinatal	6	**7**	11	**23**	0	**0**	5	**4**
	Neonatal/infant	0	**0**	2	**4**	0	**0**	26	**10**
**Cattle**	Perinatal	0	**0**	0	**0**	0	**0**	0	**0**
	Neonatal/infant	0	**0**	0	**0**	1	**2**	1	**1**

### ZooMS

ZooMS identified both specimens from Pherae to sheep; in New Halos, ZooMS identified one specimen (HA9) as sheep and one (HA5) as goat (S6 Table). In combination with the morphological criteria, the teeth used for isotopic analysis were identified as 16 sheep and 3 goats.

### δ^13^C and δ^18^O

**Fig 7:**
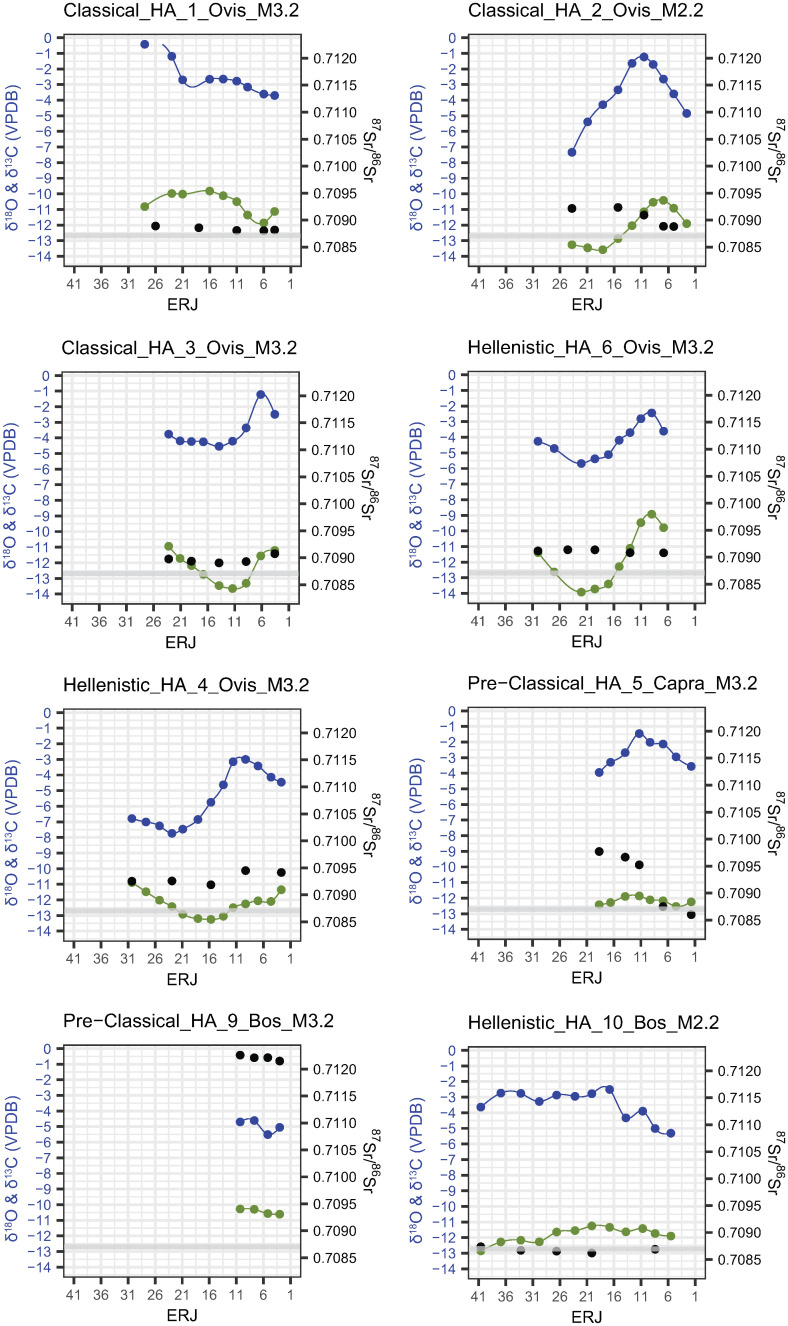
δ^18^O (blue), δ^13^C (green) and ^87^Sr/^86^Sr (black) values from enamel of mandibular second and third permanent molars in Magoula Plataniotiki. The grey band indicates the local strontium (^87^Sr/^86^Sr) baseline range.

**Fig 8:**
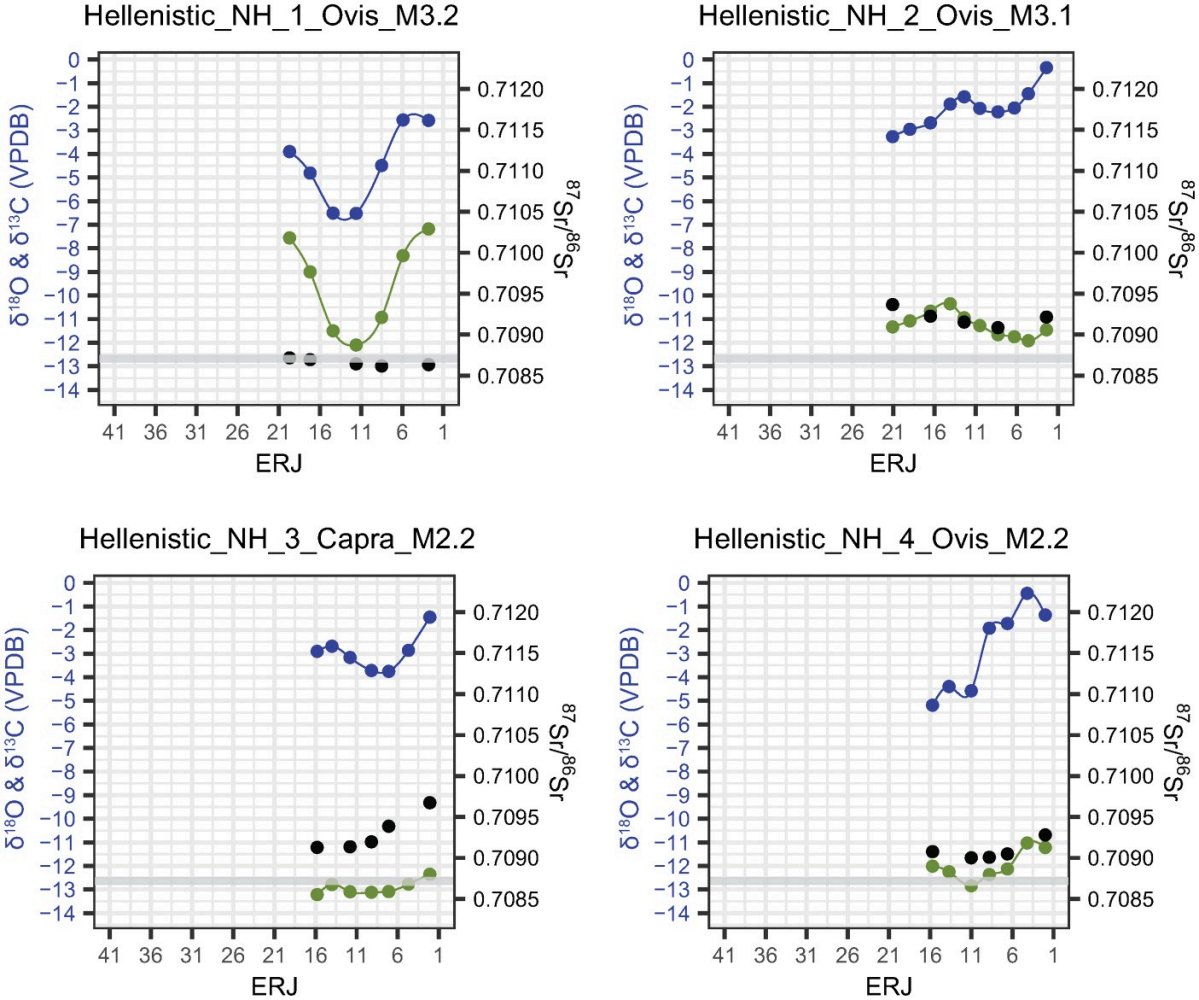
δ^18^O (blue), δ^13^C (green) and ^87^Sr/^86^Sr (black) values from enamel of mandibular second and third permanent molars in New Halos. The grey band indicates the local strontium (⁸⁷Sr/⁸⁶Sr) baseline range.

**Fig 9:**
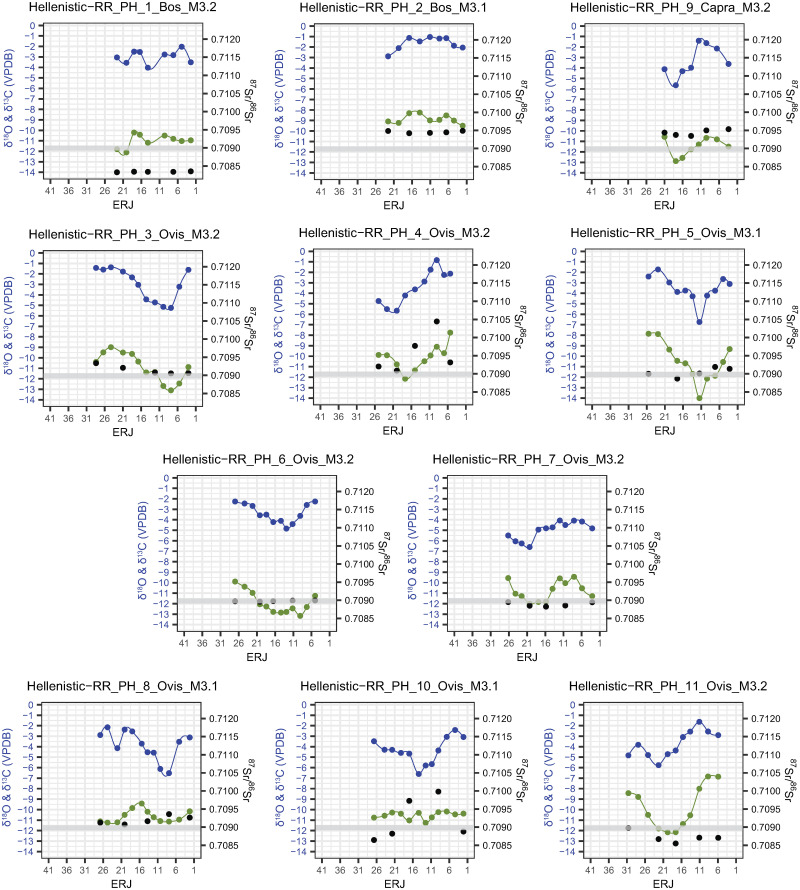
δ^18^O (blue), δ^13^C (green) and ^87^Sr/^86^Sr (black) values from enamel of mandibular second and third permanent molars in Pherae. The grey band indicates the local strontium (^87^Sr/^86^Sr) baseline range.

Incremental tooth enamel carbonate δ^13^C and δ^18^O values are shown in Figs 7–9 (Magoula Plataniotiki, New Halos, and Pherae respectively). δ^13^C and δ^18^O values are more or less correlated to each other and produce sinusoidal sequences in most of the individuals in all sites. No correlation between δ^13^C/δ^18^O and ^87^Sr/^86^Sr ratios has been identified; both flat and the few sinusoidal strontium isotope sequences coincide with sinusoidal and flat carbon and oxygen isotope sequences.

In Magoula Plataniotiki (Fig 7 and S7 Table), δ^13^C values vary from -13.9 to -8.9‰ with a mean amplitude of intra-tooth variation of 2.2‰ (from 0.3 to 5.0‰), while δ^18^O values range from -7.7 to -0.4 with a mean intra-tooth variation of 3.4‰ (from 0.9 to 6.1‰). The δ^13^C sequences of three individuals (HA9, HA5, and HA10) are flat. Τhe Δ^18^O in Classical sheep HA1, HA3, and HA6 is almost identical (3.3/3.2‰), whereas the Δ^13^C of HA6 is much higher (5‰) than the respective Δ^13^C of HA1 and HA3. HA2 and HA4 exhibit sinusoidal δ^13^C and δ^18^O sequences, albeit with a larger variation in δ^18^O values, given that the minimum δ^18^O value is more negative than for other individuals.

In Hellenistic New Halos (Fig 8 and S7 Table), the δ^13^C values of four individuals vary between -13.2 and -7.2 ‰ with a mean amplitude of intra-tooth variation of 2.3‰ (from 0.9 to 4.9‰). Sheep NH1 exhibits the least negative δ^13^C values (-7.2‰), while goat NH3 has lower Δ^13^C (0.9‰) compared to other individuals. The δ^18^O values of the same individuals range from -6.5 to -0.3‰ with a mean intra-tooth variation of 3.5‰ (from 2.3 to 4.7‰). All four individuals exhibit sinusoidal δ^18^O sequences, which are correlated with δ^13^C sequences and both δ^13^C and δ^18^O values fall roughly within the same range.

In Hellenistic Pherae (Fig 9 and S7 Table), δ^13^C values vary from -14.0 to -6.8‰ with a mean amplitude of intra-tooth variation of 3.1‰ (from 1.1 to 6.1‰), while δ^18^O values range from -6.7 to -0.8 with a mean intra-tooth variation of 3.6‰ (from 1.8 to 5.0‰). In three sheep (PH4, PH5, and PH11), the least negative δ^13^C values range between -6.8 and -7.9‰. The least negative δ^13^C ratios correspond with non-local ^87^Sr/^86^Sr ratios in PH4 and PH11, while the least negative δ^13^C ratios correspond with “local” ^87^Sr/^86^Sr ratios in PH5. In cattle PH2, the δ^13^C values are the least negative compared to the other individuals (δ^13^C between -8 and -9‰), whereas the intra-individual difference is small (Δ^13^C 1.3‰); the amplitude of δ^18^O ratios is low (Δ^18^O: 1.8‰). Similar low Δ^18^O is observed in cattle PH1 as well (2‰). Five Hellenistic sheep (PH3, PH6, PH7, PH8, and PH10) and one goat (PH9) exhibit δ^13^C more negative than -8‰; however, the Δ^13^C and Δ^18^O among these individuals vary. PH6 and PH7 share similar Δ^13^C and Δ^18^O (2.6–3.0‰ and 2.5–2.6‰ respectively) and a non-exaggerated sinusoidal patterning. Sheep PH3 has slightly higher Δ^13^C and Δ^18^O (4.1‰ and 3.9‰ respectively) whereas goat PH9 has slightly higher intra-individual δ^18^O amplitude (4.2‰) than the sheep PH3. In sheep PH8, the most negative δ^13^C values coincide with the most negative δ^18^O and least negative δ^18^O values. Finally, the sheep PH10 shows a flattened sequence of δ^13^C in 2.1–10.8mm and 19.3–26.7mm from the ERJ which is interrupted with a brief variable sequence.

### ^87^Sr/^86^Sr

#### Baseline

**Fig 10:**
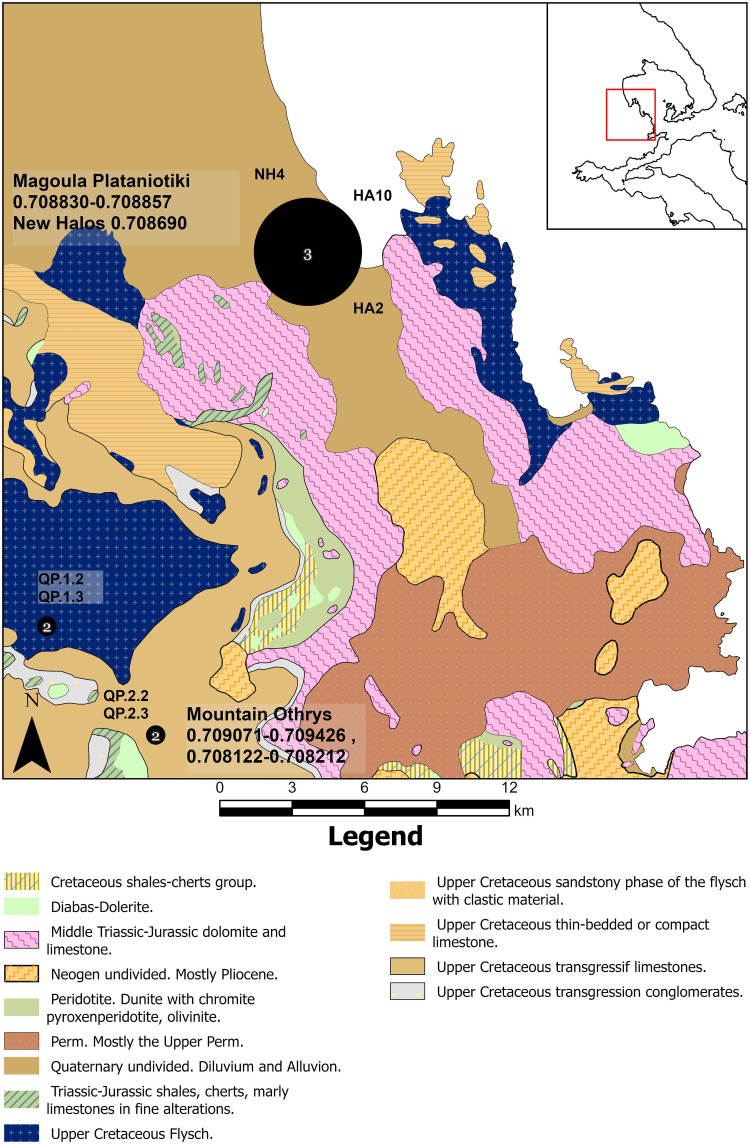
Geological map of Almyros. Prepared using material published by the Hellenic Survey of Geology and Mineral Exploration [123]. The number of samples per location is indicated in the black dot. The published information was digitized using ArcGIS Pro 3.13 by Alexandra Katevaini under a CC BY license, with permission from Alexandra Katevaini, original copyright 2023.

**Fig 11:**
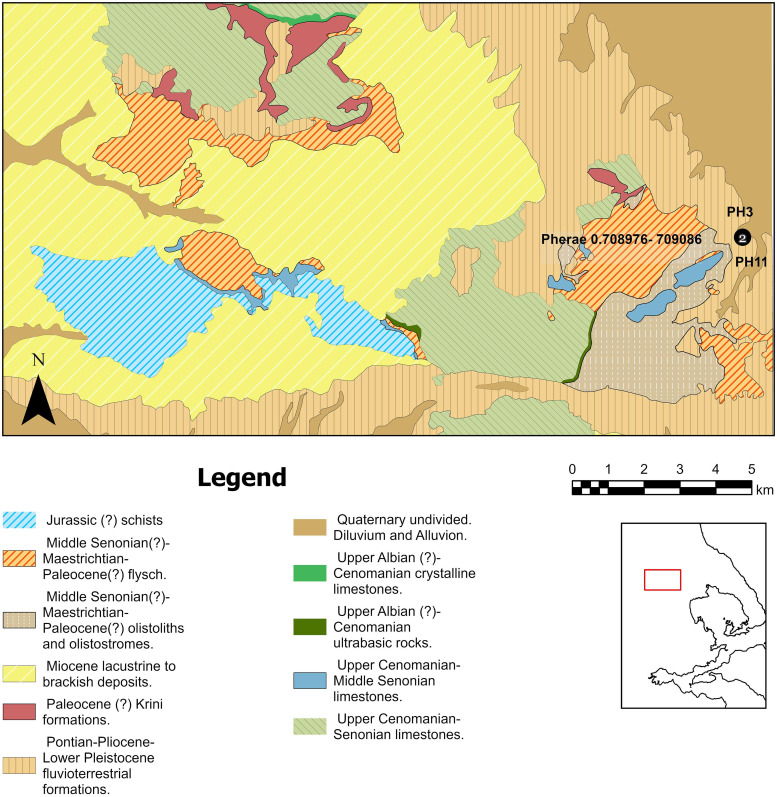
Geological map of Velestino. Prepared using material published by the Hellenic Survey of Geology and Mineral Exploration [124]. The number of samples per location is indicated in the black dot. The published information was digitized using ArcGIS Pro 3.13 by Alexandra Katevaini under a CC BY license, with permission from Alexandra Katevaini, original copyright 2023.

The bioavailable strontium isotope signature of samples QP1.2–1.3 (Upper Cretaceous flysch) in the Othrys Mountains ranges between 0.70907 and 0.70943 (Fig 10) while the values of samples QP2.2 and 2.3 (Upper Cretaceous transgressif limestones) are much depleted (0.70812–0.70821) (S8 Table). The local baseline strontium signature of Magoula Plataniotiki (ΗΑ2 and HA10) ranges between 0.70883 and 0.70886, while the respective signature in New Halos (NH4) is slightly lower (0.70869). However, the baseline strontium ratios of Magoula Plataniotiki and New Halos in the current study are higher than the measurements taken from snail shells and waters in the same geological formations in previous studies [e.g. 112]; ^87^Sr/^86^Sr from snail shells ranges from 0.7078 to 0.7080, whereas ^87^Sr/^86^Sr from water is 0.7086. In contrast, the local baseline strontium values in Pherae (PH3 and PH11) in the current study range between 0.70898 and 0.70909 (Fig 11), whereas the respective range from snail shells in Panagiotopoulou et al [112] is slightly lower (^87^Sr/^86^Sr: 0.7086–0.7088).

#### Archaeological samples

The intra-tooth variation of ^87^Sr/^86^Sr values of six out of eight individuals (pre-4^th^ century BCE HA9, Classical HA1, HA3, HA6, and Hellenistic HA4 and HA10) in Magoula Plataniotiki (Fig 7) is low (Δ^87^Sr/^86^Sr: 0.0001–0.0003). The ^87^Sr/^86^Sr signal of HA1 sheep corresponds with the baseline values, while the ^87^Sr/^86^Sr values of Classical HA3 sheep and Hellenistic HA10 cattle lie very close to the baseline values. Hellenistic sheep HA4 and HA6 have higher than the baseline values (0.70919–0.70945 and 0.70908–0.70914 respectively), while pre-4^th^ century BCE HA9 cattle has significantly higher values (0.71215–0.71226). The pre-4^th^ century BCE goat HA5 and Classical sheep HA2 show wider isotopic variability (Δ^87^Sr/^86^Sr: 0.0012 and Δ^87^Sr/^86^Sr: 0.0004 respectively) with ^87^Sr/^86^Sr values similar to the baseline corresponding with the lowest δ^18^Ο values.

As for New Halos (Fig 8), most Hellenistic sheep originate from more radiogenic geological substrates (^87^Sr/^86^Sr >0.70900) and exhibit low intra-tooth ^87^Sr/^86^Sr variation (Δ^87^Sr/^86^Sr: 0.0001–0.0003). The only exception is Hellenistic sheep NH1, whose ^87^Sr/^86^Sr ratios partially lie within the local isotopic ratio (0.70869) but minor intra-tooth variation (Δ^87^Sr/^86^Sr: 0.0001). One Hellenistic goat (NH3) shows a higher isotopic variation (Δ^87^Sr/^86^Sr: 0.0005).

Concerning Hellenistic Pherae (Fig 9), both one cattle (PH1) and one sheep (PH6) do not have any intra-tooth variation (Δ^87^Sr/^86^Sr: 0.0000). However, PH1 has distinctively lower ^87^Sr/^86^Sr values (^87^Sr/^86^Sr: 0.70833–0.70836) compared to the local baseline, whereas PH6 share similar to the local baseline values (^87^Sr/^86^Sr: 0.70897–0.70900). One cattle (PH2), one goat (PH9), and two sheep (PH5 and PH7) show slightly higher intra-tooth variation (Δ^87^Sr/^86^Sr: 0.0001–0.0002. The ^87^Sr/^86^Sr of cattle PH2 and goat PH9 is dissimilar with the baseline (^87^Sr/^86^Sr: 0.70942–0.70949 and 0.70935–0.70953, respectively), whereas the ^87^Sr/^86^Sr of Hellenistic sheep PH5 and PH7 fall within the local range. Two Hellenistic sheep (PH3 and PH8) show limited intra-tooth variation (Δ^87^Sr/^86^Sr: 0.0003) with ^87^Sr/^86^Sr values lying in the local baseline (0.70906–0.70934 and 0.70908–0.70936 respectively). Sheep PH11 and especially sheep PH4 and PH10 show higher intra-tooth variation (Δ^87^Sr/^86^Sr: 0.0014, 0.0013, and 0.0004 respectively). In PH4 and PH11, the ^87^Sr/^86^Sr that lie in the local baseline values correspond with the lowest δ^18^Ο values, whereas in PH10 the respective ^87^Sr/^86^Sr values correspond with the highest δ^18^Ο values.

## Discussion

### Inter-site variability

The new zooarchaeological evidence from Magoula Plataniotiki and New Halos confirms the apparently stable animal economy based on caprine production in addition to pig and cattle breeding. The modest rise of cattle frequency along with the slight increase of sheep size at the Hellenistic period [54] could be related to the increased urbanisation in Almiros during the Hellenistic Period as a response to the raised demand for animal products. Small-scale and semi-specialised (meat and dairy products) caprine husbandry were practised side by side in Pherae. The fact that pigs in Pherae were kept alive longer than at other sites could be related to the city’s size. A larger and potentially more diverse market could have a greater volume of people interested in the trade of pigs as breeding livestock or capital [57], whereas smaller markets could be mainly focused on selling pork meat and lard. The low frequency of pathologies on cattle remains from all sites indicates that this species was not extensively used for traction [125].

Contrary to our expectations, isotopic analysis did not suggest significant variation in mobility among the sites, despite differences in size, location, and political and economic capacity. The only distinct difference is seen in animal diet and food resourcing.

Caprines and cattle in Magoula Plataniotiki likely consumed exclusively C_3_ plants from open areas. Palynological analysis has indicated that the Almiros and Sourpi Plains were covered with arable fields, an open parkland vegetation of herbs and grasses, and small woodlets comprising deciduous oaks and other trees [11, 126]. The high degree of dental wear of cattle (pre-4th century BCE HA9) thwarts inferences regarding dietary regime. The flattened sequence of the pre-4th century BCE HA5 goat could be the result of regular foddering [80, 106, 127, 128]. The time of fodder collection and provisioning is not clear. As Isaakidou et al [106] suggested for the Late Bronze Age (late 2nd millennium BCE) Cretan goats MUM73 and RR188: “either individuals consumed an atypically less negative δ^13^C diet in winter (e.g. stored fodder harvested in summer-less negative δ^18^O values) or atypically more negative δ^13^C diet in summer (e.g. stored fodder harvested in winter-more negative δ^18^O values)”.

The diet of Hellenistic cattle HA10 was probably controlled (i.e. foddering) through the time of enamel mineralisation, and water consumption may have originated from sources that were not heavily affected by seasonal changes of temperature, most notably during the first months of enamel mineralisation (flat δ^13^C and δ^18^O sequences). Classical sheep HA1, HA3, and HA6 probably ingested water from fixed sources that were similarly affected by seasonal changes in the plains or the Othrys foothills; HA6 possibly consumed different plants or was grazing during part of the year in nearby marshes, whose salinity could elevate the δ^13^C values [e.g. 76]. HA2 and HA4 might have moved between different hydroscapes [94]; as both animals have a non-local ^87^Sr/^86^Sr signal during the winter, we may assume that at this time of the year they consumed water further inland, where the δ^18^O values are expected to be lower than those of coastal regions [94]. Alternatively, they may have experienced a more severe winter–with lower temperatures–than other animals.

In contrast, some individuals in New Halos (NH1) and Pherae (PH4, PH5, and PH11) were possibly fed with C_3_ and C_4_ plants. NH3 could either be a result of foddering or the feeding behaviour of this species; goats browse deep-rooted flora in which carbon isotope values vary less than shallow-rooted plants, usually consumed by grazers (e.g. sheep and cattle) [106, 129]. Sheep PH4 and PH11 possibly consumed C_4_ plants away from Pherae, whereas PH5 may have consumed C_4_ plants around Pherae or a region with similar geology. Cattle PH2 had a characteristically controlled diet throughout the year that possibly combined C_3_ and C_4_ plants. The low amplitude of δ^18^O ratios in cattle PH1 and PH2 could indicate water consumption from ground water sources such as water wells, shallow pools and cisterns [80, e.g. 115, 130]. However, there is no evidence of foddering for these animals, as the amplitude of δ^13^C ratios lies within the expected seasonal variation and it does not feature any flattened pattern [131]. The relatively dampened sequences could be also related to the low mineralisation rate of this species [73, 132, 133], which may cause a higher-time averaging of isotope values compared to other species.

Variation is also seen in the animals fed with C_3_ plants. Sheep PH6 and PH7 possibly consumed the available food sources around Pherae or a geologically similar region year-round, while sheep PH3 consumed a variety of plants within the same geological substrate. The feeding behaviour of goat might be relevant to the less negative δ^18^O values and intra-individual amplitude of PH9. The fact that the more negative δ^13^C values of the sheep PH8 coincide with both winter and summer months might show that it was provided with winter fodder during part of the summer. Finally, the diet of sheep PH10 did not vary much throughout the year. The flattened sequence of δ^13^C is possibly indicative of foddering, while the interpretation of the overall pattern is challenging, with one option that PH10 had a diet that was controlled relatively more strictly than the other individuals.

Given Pherae’s larger size, and consequently larger animal population, we could assume that feeding strategies of animals were more diverse at this site. It is possible that livestock had access to either millet (C_4_) or pastures on the shores of the Pagasetic Gulf, where C_4_ plants could have grown. However, provided that the faunal assemblages of Magoula Plataniotiki and Pherae represent a time-span of at least three centuries, whether feeding strategies changed over time or were diversified throughout this period cannot be quantified. The fact that similar mobility patterns were observed in three dissimilar sites implies that animal husbandry practices did not vary significantly in the wider region, regardless of the town or city that reared the animals and exploited their products.

Whether the surplus from animal husbandry was used to cover the demand for grain, as Haagsma has argued for New Halos, remains unclear [31]. This relatively large town possibly had extra requirements for animal products due to both its expansiveness and its high demand for labour mobilization to support the building of fortifications and houses in a short period. Nevertheless, the limited zooarchaeological and isotopic evidence suggests small-scale animal production, which implies that this type of animal management may have been locally unsustainable, as compared to the much smaller site of Magoula Plataniotiki. We do not support animal husbandry per se being the reason that New Halos was abandoned, but it is possible that instability disrupted animal husbandry which in turn had a negative impact on the local economy in the long-term. On the contrary, Magoula Plataniotiki and Pherae managed to survive despite the constant instability in southern Thessaly, suggesting that warfare had not the same impact in the local economies during the Hellenistic Period. A larger dataset could provide more information on the importance of animal husbandry in the local economies.

Contrary to the current study, the few studies in Greece that have examined animal management and mobility using isotope analysis have revealed heterogeneity in the degree of mobility among different species. Late Bronze Age goats in Crete showed more restricted mobility from lowland to highland areas compared to some sheep that showed seasonal vertical movements [106]. Additionally, Late Neolithic (5500±5000/4900 cal BCE) sheep in Makriyalos, Greek Macedonia, possibly grazed locally when young, whereas cattle pastured more widely [128]. The absence of any discernible difference between taxa in the current research, apart from the flattened sequences in cattle, could be indicative of mixed herds; a few goats were included in herds to guide the sheep, which remains a common practice today [134]. Similar caprine management has been observed in the neighbouring Hellenistic sites of Kastro Kallithea and Pharsalos [21], which indicates homogeneity in caprine husbandry in the wider region of southern Thessaly.

### Mobile or sedentary management?

The representation of all age groups including perinatal individuals indicates that at least part of the flocks remained around the site year-round [58] and were possibly penned inside the settlements [59]. ^87^Sr/^86^Sr ranges similar to the current study have been reported from other geological substrates in the region, in both the Othrys Mountains (0.708160–0.708607; 0.708441; 0.709384) and neighbouring lowland areas, e.g. Kastro Kallithea (0.708240–0.709030) and Pharsalos (0.7078–0.7090) [20, 112]. As such, identifying vertical movements becomes complicated.

^87^Sr/^86^Sr values provide further evidence for sedentary animal husbandry year-round in all sites. Some animals were reared in fixed pastures around the site or in geologically similar regions (e.g. HA1, HA10, NH1, PH5, PH6, PH7), while cattle (HA9 and PH1) were sedentary but reared in distinctively different pasturelands. Most individuals moved either close to the settlement or a region with identical geology (HA3, PH3, and PH8), or in pastures further away in a similar altitude (HA4 HA6, NH2, NH4, PH2, and PH9).

However, several others (HA2, HA5, PH4, PH10, and PH11) moved seasonally between the site (or a region with similar strontium isotope signatures) and locations with different geology. The pre-4^th^ century BCE goat (HA5) was raised during the spring and summer in one region (less negative δ^18^O) and translocated near the site or in a geologically similar area, between autumn and winter (more negative δ^18^O ratios). The Classical sheep HA2 pastured in winter and spring in another location and moved to the site or to a geologically similar area in autumn. The distribution and amplitude of δ^18^O values suggest that these animals crossed areas with similar altitude and seasonal temperature variation. NH3 was mobile but moved towards areas away from New Halos, which suggests higher mobility during the first year of the animal’s life. That movement likely occurred during the cooler months of the year. Sheep PH4 possibly pastured nearby the site or in a location with ^87^Sr/^86^Sr values during the winter months, moved to another location during the spring and summer, and returned to Pherae or a geologically similar region in the autumn. In contrast, PH10 probably pastured around Pherae or in a geologically identical region in the summer months, moved to a region with different geology in the winter, and returned back in the summer. PH11 likely spent the autumn months around Pherae or in a geologically similar region and moved to another region from winter onwards.

No significant differences were observed in mobility patterns among taxa, sites, and periods. The lack of an inverse seasonal pattern in δ^13^C and δ^18^O, as seen in the neighbouring towns of Pharsalos and Kastro Kallithea [21] and on prehistoric Crete [106], implies no vertical movements of herds between seasons (transhumance) [e.g. 69] as suggested by Reinders and Prummel [7].

Based on the above observations, animal management seemed to be diverse and various grazing lands were probably used in all three locations. Degree of mobility likely varied among individuals, as did the season that an animal pastured near a settlement. The salty marshes of the Almiros Plain and the foothills of the Othrys Mountains [25], as well as the Greater Thessalian Plain and the surrounding hills [28], could provide rich pasture for the herds. Some animals could have herded on these plains within the chora (rural hinterland) year-round, while others possibly moved outside the chora daily or seasonally. Networks of farmhouses around Halos [135] and Pherae [46] must have participated in animal husbandry, but their precise role remains to be examined with further research.

Combined isotopic results in Pherae revealed that some animals had a “local” strontium signature during the winter months while others had one in the summer months. Whether these individuals were raised near Pherae or a geologically similar region during different times of the year, it is plausible that different herds had access to poleis’ (cities) territories at different times of the year. Such complex animal management would require coordination with the local or regional authorities (e.g. arbitration between Chyretiai and another city [*SEG* 45: 588]) to negotiate over grazing lands [136]. Epigraphic evidence from the Hellenistic period records several conflicts between towns over grazing rights (e.g. *IG*, IX, 2, 522; *SEG*, 13, 391; GHW03078; *IG*, IX, 2, 89; FD III, iv, 355[3]). At the same time, the regulation of grazing areas could diminish the risk of raids and animal theft [see 137, 138]. These examples imply that land management for herding activities was an important aspect of political negotiations in the wider region and that animal husbandry (and animal mobility) may have been regulated institutionally. However, a larger dataset of isotopic values is needed to test this hypothesis.

Herders could drive flocks in neighbouring poleis thanks to epinomia, a privilege given by the polis, that allowed herding in both the region of the beneficiary’s origin and the chora of the polis granted this privilege. Given that Pherae and New Halos are among the communities in Thessaly that have recorded epinomia allotments [139], it seems that the constant conflicts among leagues/empires during these periods [23, 140–143] were ameliorated by such privileges. The intra-site variation in ^87^Sr/^86^Sr values and the overall mobility patterns suggest that various pasturelands were available around the sites. Such variation could be a result of epinomia, because the beneficiary had access to numerous public lands associated with the polis [144]. Alternatively, it is plausible that separate co-existing farms at each site had access to different pasturelands. In addition to the poleis, sanctuaries could also give permission for grazing on their land (e.g. the Classical Temple of Athena Alea in Tegea [IG V.2 in Osborne [145].

Considering the importance of animal sacrifice in public rituals in ancient Greece, we could assume that part of the faunal remains represent food consumed during public events [13]. Although ancient sources [146–148] hint that from the 4^th^ century BCE onwards, part of the population, likely the elite, consumed meat more regularly [149], meat consumption was still generally associated with sacrifices and other public occasions [150, 151]. Therefore, it is likely that sanctuaries and public festivals obtained their food resources from different herds, owned by either several farms or a few wealthy individuals who benefited from epinomia. More epigraphic evidence from the examined sites could shed light on the impact of epinomia and sacrifices in animal management and complement the limited zooarchaeological and isotopic data.

### From mobility to scale of production

The absence of a strictly caprine-oriented husbandry implies that all main domesticates played important roles in local and regional economies. Additionally, kill-off patterns show that caprines and cattle were kept for both their primary and secondary products [60] in Magoula Plataniotiki and New Halos, while there is no evidence of either intensive slaughtering of young pigs or intentional yield maximisation, because animals were slaughtered in various age stages. As animal production was not conducted on a large scale, we can assume that extended animal movement was not generalised.

Some of the animals that showed a greater degree of mobility either were foddered when the available pasture was insufficient to cover their nutritional needs or obtained their food from different sources. Regarding the former interpretation, the relatively small “Hellenistic size” of livestock [54, 55] does not provide evidence for intensive foddering and consequently intensive animal management. Herders did not attempt (or were not able) to improve the productivity of their livestock, which implies that animal husbandry was not on a large scale overall. Alternatively, the ingestion of food from different sources could be linked to a greater degree of mobility; animals were moving to different locations and consumed locally sourced food.

The fact that transhumance movements have not been identified in the studied material does not necessarily mean that they were not practised at all in the wider area. Bishop and her colleagues found that one goat could be part of a transhumant herd in neighbouring Kastro Kallithea, while some animals in Magoula Plataniotiki and New Halos moved seasonally, which suggests that skilled herders were required to coordinate flocks’ movements [20].

The variation in mobility patterns in Magoula Plataniotiki, New Halos, and Pherae could reflect a diversity in the intensity of animal husbandry strategies; households could raise animals locally for subsistence [57, 152], whilst local elites could own large herds with mobile animals and thus target profit [10]. Animal husbandry was not only a profitable activity; it was also a chance for the Thessalian elite to demonstrate their wealth and consolidate their status (e.g. Jason of Pherae [17] and Thessalian oligarchs in general [22]). The elite considered land and livestock ownership the most socially acceptable form of wealth in the Greco-Roman world, in comparison to trade or the manufacturing of goods [10]. Land cultivation and animal breeding were considered dependent on the gods’ will to provide rain to plants and fertility to animals [see 10, 153–155]. Therefore, the elite considered owning livestock a god-blessed activity and consequently a means to highlight virtues.

### Southern Thessaly and the agropastoral debate

So far evidence from the three examined sites suggests a mixed animal management in which a small-scale, sedentary, and possibly non-specialised animal rearing was practised side-by-side with larger-scale, mobile, and semi-specialised production. Contrary to zooarchaeological data, which suggest a small-scale sedentary economy, isotopic evidence from New Halos and Magoula Plataniotiki imply a complex animal management that entailed individuals being herded in various locations, possibly for different purposes. Despite this complexity, no altitudinal movements between seasons (transhumance) were identified, which could indicate that animal economies were not oriented towards specialised mobile pastoralism. Zooarchaeological evidence coordinates with isotopic evidence in Pherae, as neither reveals an exclusively subsistence or market economy. As with animals from Magoula Plataniotiki and New Halos, Pheraean animals were exploited in different ways. Bishop’s isotopic work in the neighbouring Hellenistic sites of Kastro Kallithea and Pharsalos reveals similar diversity in animal management, which further supports the argument that the “mixed model” prevailed in southern Thessaly during the Classical antiquity [20, 21]. Although Thessaly was considered rich in grains and livestock, textual evidence (*SEG* 9:2) attests to a food crisis due to drought around 320 BCE in Larissa, Atrax, and Meliboia [25] and supply disruption due to war in 171 BCE [143]. Diverse animal management could have contributed to risk management [151].

The landscape could support a complex system of animal husbandry; small plains with hills and the gradual foothills of the Othrys accommodated daily and seasonal movements, while the Greater Thessalian Plain provided the animals with access to more food resources. The absence of evidence for extensive livestock exploitation indicated that herding alone did not cause the vegetation change in the Othrys Mountains and on the Almiros Plain, seen in the palynological record [11, 12]; timber for the construction of fortifications, houses, and ships may have played an important role as well. Although textual evidence implies herding and seasonal mobility in the Othrys Mountains in antiquity, interpretation should be cautious seeing as the relevant texts are poems and myths [156–158]. The fact that some animals were herded seasonally in the Othrys Mountains, does not necessarily mean that animal husbandry was entirely based on transhumant pastoralism; zooarchaeological and isotopic analyses here, on a small sample size do not provide evidence of transhumance.

To sum up, zooarchaeological analysis suggests that some animals were possibly kept in and/or around the sites year-round. Isotopic results indicate sedentary husbandry, as some individuals were reared in the same geology in the first years of their lives with limited or no mobility, whilst other animals showed greater mobility. Finally, some individuals moved seasonally across regions in the same altitude at different times of the year. No large-scale transhumance patterns were identified. Variation in mobility implied variation in the intensity of animal husbandry. Some herds could have been exploited intensively for subsistence, while others for markets. Moreover, it is also possible that plurality in mobility patterns could reveal movements regulated by benefits granted via epinomia and/or the coexistence of various farms within sites’ hinterlands. Most of the animals consumed fresh food, whilst a few were foddered. Overall, no remarkable differences among sites and periods were identified, which implies the presence of similar zootechniques. Bringing all of the available evidence together, the proposed model of animal husbandry for the examined region during the 4th-1st centuries BCE fits the “mixed model” of combined sedentary, non-specialised and mobile, semi-specialised livestock management. The continuous warfare likely impacted animal husbandry and prevented the large-scale production. Such a mixed strategy could meet the needs of different segments of societies for both subsistence and the market and could decrease the risk of a shortage in animal products.

Further work is required to better define the animal husbandry practices in Classical and Hellenistic Thessaly, as the results here are based on limited sample size of only 23 individuals across the three sites. Larger datasets including sieved samples and material from upland sites and farmhouses will provide more information about animal management, mobility, and connections between the highlands and lowlands. Moreover, because the geology of Thessaly is complex, additional modern samples will increase the resolution of the baseline map and make it possible to identify the location of past animal movements. Finally, comparative work with contemporary sites in Greece and the eastern Mediterranean plains is necessary to understand how the extended instability of the wider region affected different animal husbandry regimes.

## Supporting information

S1 File Protocol for the baseline sampling. Dimitris Filioglou and Silvia Valenzuela-Lamas.
(DOCX)

S1 Text ZooMS analysis. Samantha Presslee.
(DOCX)

S2 Text Radiocarbon measurement. Sanne Palstra.
(DOCX)

S3 Text Strontium isotope analysis (archaeological and baseline samples). Leopoldo D. Pena.
(DOCX)

S4 Text Carbon and oxygen stable isotope analysis. William P. Patterson and Sandra Timsic.
(DOCX)

S5 Text Official permission. (DOCX)

S1 Table Calibrated dating results. The 95.4% (2σ) probability range demonstrated in the figure is based on the 14C measurement result. The date range indicates the time periods matching the measured 14C value at this level of probability.
(DOCX)

S2 Table Main domesticates’ frequency in Magoula Plataniotiki, New Halos, and Pherae in number of identified specimens (NISP) based on diagnostic zones (DZ) [1]. (DOCX)

S3 Table Sheep/goat ageing data in Magoula Plataniotiki, New Halos, and Pherae based on mandibular tooth wear and eruption after Payne [2, 3]. Deciduous fourth premolar = dP4. Permanent fourth premolar = P4, first molar = M1, second molar = M2, third molar = M3.
(DOCX)

S4 Table Pig ageing data of lassical Magoula Plataniotiki, Hellenistic Magoula Plataniotiki, and Pherae based on epiphyseal fusion after Zeder Lemoine and Payne [4]. (*) The three fusing distal tibiae and one fusing distal metacarpal are listed under the fused category.
(DOCX)

S5 Table Cattle ageing data of Classical Magoula Plataniotiki, Hellenistic Magoula Plataniotiki, and Pherae based on epiphyseal fusion after Reitz and Wing [5]. (*) The single fusing distal tibia is listed under the fused category. Loose epiphyses are excluded.
(DOCX)

S6 Table ZooMS collagen peptide mass fingerprint spectra of NH2 (00190–7), NH3 (00190–6), PH10 (00190–2), and PH11 (00190–1). The results were compared to a database of published refences markers [6–8]. The annotated peaks represent the markers used to make the identifications, specifically marker 3033.4 for sheep and 3093.4 for goat.
(DOCX)

S7 Table Isotopic data (δ^13^C, δ^18^O, and ^87^Sr/ ^86^Sr) recorded from caprine and cattle mandibular third and second molar enamel according to distance from the enamel-root junction (ERJ) (mm). 2σ = 2 standard deviation.
(DOCX)

S8 Table Strontium isotopic ratios (^87^Sr/^86^Sr) obtained on modern tree leaves and animal bones from different geologic formations. (DOCX)
