# Supplementary material for: Where did the herds go? Combining zooarchaeological and isotopic data to examine animal management in ancient Thessaly (Greece)
Source: PLoS One. 2024 Oct 22;19(10):e0299788. doi: 10.1371/journal.pone.0299788 (PMC11495569; doi:10.1371/journal.pone.0299788)
Supplement: S1 File — Dimitris Filioglou and Silvia Valenzuela-Lamas. (DOCX) [file pone.0299788.s001.docx]

**S1 Protocol. Protocol for the baseline sampling.** Dimitris Filioglou and Silvia Valenzuela-Lamas.

Type of plant:

Tree leaves from oaks (e.g. Kermis oak), olive trees and/or pines because they have deep roots.

The bigger and older a tree is the better.

Choose plants of similar height/age.

Location of sampling:

Far away from motorways, houses, industry and rivers that may bring pollution to the sample.

“Virgin” geological layers.

In the middle of a geological layer, not in the margins in order to avoid contamination.

If possible, from sites with known shepherd use.

On site sampling:

Take pictures of the location you are sampling.

Keep track of the coordinates of the sampling location (GPS).

Assign a unique name/number/code of each sample.

Take pictures of each sample.

Record (on the envelope, on a notepad or on another device) the geological layer you just sampled together with the GPS coordinates.

Record the date of collection to know the season.

Storing:

Put each leaf sample in a paper-envelope. Never use a plastic bag.

Indicate the name of the sample on the envelope.
