# Supplementary material for: Where did the herds go? Combining zooarchaeological and isotopic data to examine animal management in ancient Thessaly (Greece)
Source: PLoS One. 2024 Oct 22;19(10):e0299788. doi: 10.1371/journal.pone.0299788 (PMC11495569; doi:10.1371/journal.pone.0299788)
Supplement: S1 Text — Samantha Presslee. (DOCX) [file pone.0299788.s002.docx]

Supporting Information- Text

**S1 Text. ZooMS analysis.** Samantha Presslee.

The bone samples were demineralised in 0.6 M HCl for 72 hours at 4 °C, washed in 0.1 M sodium hydroxide to remove possible humic acid contamination, followed by several washes in 50mM ammonium bicarbonate (AmBic). 200 µl AmBic was added to the samples and they were gelatinised for 1 hour at 65 °C, which allows the available collagen to solubilise into solution. The sample was then split; 100 µl of the supernatant was pipetted into a new microfuge tube while the remaining 100 µl, containing the demineralised bone sample, was stored at -20 °C for further analysis if needed. The 100 µl supernatant was digested overnight with the addition of 1 µl of 0.4 µg trypsin (Promega UK) and heated at 37 ˚C for ~18 hours. Digestion was stopped with the addition of 1 µl 5% TFA, and the collagen peptides were purified using 100 µl C18 resin ZipTip pipette tips before being eluted in 100 µl 50:50 ACN: 0.1% TFA in distilled water.

The samples were spotted in triplicate onto a Bruker MALDI plate, mixing 1 µl of the sample with 1 µl matrix solution (α-cyano-hydroxycinnamic acid). MALDI-ToF-MS (Matrix Assisted Laser Desorption Ionisation- Time of Flight Mass Spectrometry) analysis was carried out using a Bruker Ultraflex III mass spectrometer, and the mass spectra were analysed using the software mMass [1]. An averaged mass spectrum was created from the three replicates and peaks were picked using a s/n ratio set at 4, The mass spectra were also cropped between the m/z range of 800-3500. The resulting peaks were compared to published markers of sheep or goat [2].

# **References**

1. Strohalm M, Kavan D, Novák P, Volný M, Havlícek V. mMass 3: A Cross-Platform Software Environment for Precise Analysis of Mass Spectrometric Data. Anal Chem [Internet]. 2010;82(11):4648–4651. Available from: https://doi.org/10.1021/ac100818g

2. Buckley M, Kansa SW, Howard S, Campbell S, Thomas-Oates J, Collins M. Distinguishing between archaeological sheep and goat bones using a single collagen peptide. J Archaeol Sci [Internet]. 2010;37(1):13–20. Available from: http://dx.doi.org/10.1016/j.jas.2009.08.020
