# Supplementary material for: Where did the herds go? Combining zooarchaeological and isotopic data to examine animal management in ancient Thessaly (Greece)
Source: PLoS One. 2024 Oct 22;19(10):e0299788. doi: 10.1371/journal.pone.0299788 (PMC11495569; doi:10.1371/journal.pone.0299788)
Supplement: S2 Text — Sanne Palstra. (DOCX) [file pone.0299788.s003.docx]

Supporting Information- Text

**S2 Text. Radiocarbon measurement.** Sanne Palstra

A 30mg sample of tooth dentine was analysed at the Centre for Isotope Research (CIO) of the University of Groningen, The Netherlands, following Dee et al [1]. 14C ages (in yrBP) were calibrated to calendar years with the software program “OxCal, version 4.4” [2] using a calibration curve “IntCal20” [3]. Note: ‘Yld (%)’ is the collagen yield relative to the pretreated bone sample. δ^13^C is measured relative to ‘VPDB’ and δ^15^N is measured relative to ‘AIR’. The reported uncertainties in the measurement results include variations in the analysis of carbon isotopes as observed between subsamples of the same sample material (of homogeneous isotope composition and similar sizes). These are variations in the chemical pre-treatment, combustion and isotope measurement.

# **References**

1. Dee MW, Palstra SWL, Aerts-Bijma AT, Bleeker MO, de Bruijn S, Ghebru F, et al. Radiocarbon Dating at Groningen. Radiocarbon [Internet]. 2020;62(1):63–74. Available from: https://doi.org/10.1017/RDC.2019.101

2. Bronk Ramsey C. Bayesian analysis of radiocarbon dates. Radiocarbon [Internet]. 2009;51(1):337–60. Available from: https://doi.org/10.1017/S0033822200033865

3. Reimer P, Austin W, Bard E, Bayliss A, Blackwell P, Bronk Ramsey C, et al. The IntCal20 Northern Hemisphere Radiocarbon Age Calibration Curve (0–55 cal kBP). Radiocarbon [Internet]. 2020;62(4):725–57. Available from: doi:10.1017/RDC.2020.41
