# Supplementary material for: Where did the herds go? Combining zooarchaeological and isotopic data to examine animal management in ancient Thessaly (Greece)
Source: PLoS One. 2024 Oct 22;19(10):e0299788. doi: 10.1371/journal.pone.0299788 (PMC11495569; doi:10.1371/journal.pone.0299788)
Supplement: S3 Text — Leopoldo D. Pena. (DOCX) [file pone.0299788.s004.docx]

Supporting Information- Text

**S3 Text. Strontium isotope analysis (archaeological and baseline samples).** Leopoldo D. Pena.

Samples for strontium isotope analyses were further processed at the Laboratori d’Isòtops Radiogènics i Ambientals (LIRA) at Universitat de Barcelona. Strontium purification is then carried out using standard procedures with Triskem® Sr-Spec resin, with high specificity for Sr^2+^ cations that achieves an excellent separation from interfering element Rb. Following sample purification, Sr isotope ratios were determined by multicollector inductively coupled mass spectrometry on a Nu Instruments (Wrexham, UK) Plasma 3 MC-ICPMS at the University of Barcelona (CCiT-UB). For strontium isotope analysis a mathematical correction of the ^87^Rb isobaric interference on ^87^Sr was performed from the signal measured for ^85^Rb assuming a ^87^Rb/^85^Rb value of 0.3857 [1]. An iterative mathematical correction of the ^86^Kr isobaric interference on ^86^Sr signal [2] was carried out using the measured ^83^Kr signal and assuming a ^86^Kr/^83^Kr ratio of 1.503 [1]. A total of 5 iterations was enough to obtain a constant value for the mass bias factor and the ^87^Sr/^86^Sr ratio. An internal correction of mass bias was carried out by measuring the ^88^Sr/^86^Sr ratio and using the exponential model [3] to correct ^86^Sr/^88^Sr = 0.1194 for mass bias correction. The reference material SRM 987 (NIST) was also analysed bracketing the samples using a value of 0.710249 for the ^87^Sr/^86^Sr in the SRM 987 [4–6]. Samples and standards were matched in matrix and concentration. The yield of the chromatographic separation of Sr and Rb was verified by measuring [Sr]/[Rb] ratio >1000, thus leading to a negligible effect of the Rb interference correction on the accuracy of results. External analytical reproducibility during the session was ± 0.000018 (2σ, n=19).

# **References**

1. de Laeter J, Böhlke J, De Bièvre P, Hidaka H, Peiser H, Rosman K, et al. Atomic weights of the elements. Review 2000 (IUPAC Technical Report). Pure Appl Chem [Internet]. 2003;75(6):683–800. Available from: https://doi.org/10.1351/pac200375060683

2. Weber M, Lugli F, Hattendorf B, Scholz D, Mertz-Kraus R, Guinoiseau D, et al. NanoSr – A New Carbonate Microanalytical Reference Material for In Situ Strontium Isotope Analysis. Geostand Geoanalytical Res [Internet]. 2020;44(1):69–83. Available from: https://doi.org/10.1111/ggr.12296

3. Wombacherab F, Rehkämper M. Investigation of the mass discrimination of multiple collector ICP-MS using neodymium isotopes and the generalised power law. J Anal At Spectrom [Internet]. 2003;18(11):1371–5. Available from: https://doi.org/10.1039/B308403E

4. Azmy K, Veizer J, Wenzel B, Bassett MG, Copper P. Silurian strontium isotope stratigraphy. Geol Soc Am Bull [Internet]. 1999;111(4):475–483. Available from: https://doi.org/10.1130/0016-7606(1999)111%3C0475:SSIS%3E2.3.CO;2%0A

5. Ando A, Khim BK, Nakano T, Takata H. Chemostratigraphic documentation of a complete Miocene intermediate-depth section in the Southern Ocean: Ocean Drilling Program Site 1120, Campbell Plateau off New Zealand. Mar Geol [Internet]. 2011;279(1–4):52–62. Available from: https://doi.org/10.1016/j.margeo.2010.10.012

6. Cramer BD, Munnecke A, Schoﬁeld DI, Haase KM, Haase-Schramm A. A Revised 87Sr/86Sr Curve for the Silurian: Implications for Global Ocean Chemistry and the Silurian Timescale. J Geol [Internet]. 2011;119(4):335–349. Available from: https://doi.org/10.1086/660117
