# Supplementary material for: Where did the herds go? Combining zooarchaeological and isotopic data to examine animal management in ancient Thessaly (Greece)
Source: PLoS One. 2024 Oct 22;19(10):e0299788. doi: 10.1371/journal.pone.0299788 (PMC11495569; doi:10.1371/journal.pone.0299788)
Supplement: S4 Text — William P. Patterson and Sandra Timsic. (DOCX) [file pone.0299788.s005.docx]

Supporting Information- Text

**S4 Text. Carbon and oxygen stable isotope analysis.** William P. Patterson and Sandra Timsic**.**

Samples for carbon and oxygen isotope analyses were further processed at the Saskatchewan Isotope Laboratory, Department of Geological Sciences Department (University of Saskatchewan, Canada) and the Petrology and Geochemistry Department of the Spanish National Research Council (Granada, Spain). Carbonate samples were roasted in a vacuum oven at 200°C for 1 hour to remove water and volatile organic contaminants that may confound stable isotope values of carbonates. Stable isotope values were obtained using a Finnigan Kiel-IV carbonate preparation device directly coupled to the dual inlet of a Finnigan MAT 253 isotope ratio mass spectrometer. 20-50 micrograms of carbonate were reacted at 70^o^C with 3 drops of anhydrous phosphoric acid for 420 seconds. The CO_2_ evolved was then cryogenically purified before being passed to the mass spectrometer for analysis. Isotope ratios were corrected for acid fractionation and ^17^O contribution using the Craig correction, and they were reported in per mil notation relative to the VPDB scale. Data were directly calibrated against the international standard NBS-19 that has the following values: δ^13^C = 1.95‰ VPDB and δ^18^O = -2.20‰ VPDB. Precision/accuracy of data were monitored through routine analysis of NBS-19. Precision/accuracy of δ^13^C and δ^18^O are 0.05‰ and 0.11‰, respectively (n = 25). Actual sample errors may be greater than these due to sample heterogeneity.
