# Supplementary material for: Where did the herds go? Combining zooarchaeological and isotopic data to examine animal management in ancient Thessaly (Greece)
Source: PLoS One. 2024 Oct 22;19(10):e0299788. doi: 10.1371/journal.pone.0299788 (PMC11495569; doi:10.1371/journal.pone.0299788)
Supplement: S5 Text — (DOCX) [file pone.0299788.s006.docx]

Supporting Information- Text

**S5 Text. Official permission.**

Permission to carry out the scientific analyses of the materials from Magoula Plataniotiki, New Halos, and Pherae was issued by the Ephorate of Magnesia, Hellenic Ministry of Culture and Sports (permits: Magoula Plataniotiki - ΥΠΠΟΑ/ΓΔΑΠΚ/ΕΦΑΜΑΓ/ΤΠΚΑΜ/624973/445710/5783/2321/18-12-19; New Halos - ΥΠΠΟΑ/ΓΔΑΠΚ/ΔΙΠΚΑ/ΤΕΕΑΕΙ/704840/502134/14251/2188/02-01-2020; Pherae - ΥΠΠΟΑ/8546/19-01-2021). The samples are stored in a government-controlled storage facility in modern-day Almiros and Velestino, Greece, and are not publicly accessible. For research purposes, access to the materials can be sought from the Greek Ministry of Culture, with support from the excavations’ co-directors: Dr Vaso Rondiri (Magoula Plataniotiki and New Halos) and Dr. Roula Doulgeri-Intzesiloglou and Polixeni Arachoviti (Pherae). The study complied with all relevant regulations.
