# Supplementary material for: Where did the herds go? Combining zooarchaeological and isotopic data to examine animal management in ancient Thessaly (Greece)
Source: PLoS One. 2024 Oct 22;19(10):e0299788. doi: 10.1371/journal.pone.0299788 (PMC11495569; doi:10.1371/journal.pone.0299788)
Supplement: S1 Table — The 95.4% (2σ) probability range demonstrated in the figure is based on the 14C measurement result. The date range indicates the time periods matching the measured 14C value at this level of probability. (DOCX) [file pone.0299788.s007.docx]

Supporting Information- Tables

| **Sample name** | **Dated material** | **Lab-ID** | **F^14^C** | **± 1-sigma** | **^14^C Age (yrBP)** | **± 1-sigma** |
| --- | --- | --- | --- | --- | --- | --- |
| HA5 | Collagen | GrM-29118 | 0.7355 | 0.0019 | 2468 | 22 |

| **Sample name** | **Yld (%)** | **%C** | **%N** | **C/N** | **δ^13^C (‰;IRMS)** | **± 1-sigma** | **δ^15^C (‰;IRMS)** | **± 1-sigma** |
| --- | --- | --- | --- | --- | --- | --- | --- | --- |
| HA5 | 1.2 | 40.6 | 15.3 | 3.1 | -20.37 | 0.15 | 6.72 | 0.30 |

| **Sample name** | **Lab-ID** | **From** | **To** | **Probability (%)** |
| --- | --- | --- | --- | --- |
| HA5 | GrM-29118 | 760 BC | 425 BC | 95.4 |


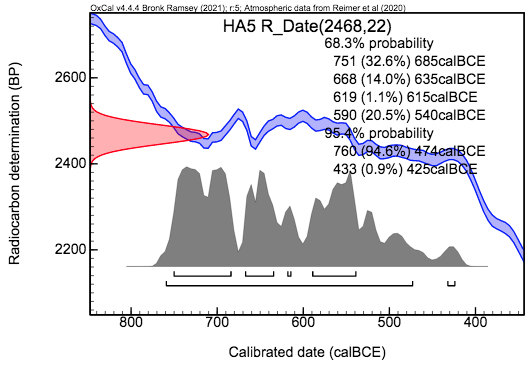


**S1 Table. Calibrated dating results.** The 95.4% (2σ) probability range demonstrated in the figure is based on the 1^4^C measurement result. The date range indicates the time periods matching the measured ^14^C value at this level of probability.
