# Supplementary material for: Where did the herds go? Combining zooarchaeological and isotopic data to examine animal management in ancient Thessaly (Greece)
Source: PLoS One. 2024 Oct 22;19(10):e0299788. doi: 10.1371/journal.pone.0299788 (PMC11495569; doi:10.1371/journal.pone.0299788)
Supplement: S2 Table — (DOCX) [file pone.0299788.s008.docx]

Supporting Information- Tables

| **Taxa** | **Magoula Plataniotiki** | | | | **New Halos** | | **Pherae** | |
| --- | --- | --- | --- | --- | --- | --- | --- | --- |
|  | **Classical** | | **Hellenistic** | | **Hellenistic** | | **Hellenistic** | |
|  | **DZ** | **%** | **DZ** | **%** | **DZ** | **%** | **DZ** | **%** |
| Caprines | 139 | **56%** | 96.5 | **55%** | 98 | **65%** | 534.5 | **63%** |
| Pig | 78.5 | **31%** | 42 | **24%** | 11 | **7%** | 235 | **28%** |
| Cattle | 32 | **13%** | 37 | **21%** | 41 | **28%** | 81 | **9%** |
| Total | 249.5 | **100%** | 175.5 | **100%** | 150 | **100%** | 849 | **100%** |

**S2 Table. Main domesticates’ frequency in Magoula Plataniotiki, New Halos, and Pherae in Number of Identified Specimens (NISP) based on Diagnostic Zones (DZ)** **[1]*.***

# **References**

1. Watson JPN. The estimation of the relative frequencies of mammalian species: Khirokitia 1972. J Archaeol Sci. 1979;6:127–37.
