# Supplementary material for: Where did the herds go? Combining zooarchaeological and isotopic data to examine animal management in ancient Thessaly (Greece)
Source: PLoS One. 2024 Oct 22;19(10):e0299788. doi: 10.1371/journal.pone.0299788 (PMC11495569; doi:10.1371/journal.pone.0299788)
Supplement: S3 Table — Deciduous fourth premolar = dP4. Permanent fourth premolar = P4, first molar = M1, second molar = M2, third molar = M3. (DOCX) [file pone.0299788.s009.docx]

Supporting Information- Tables

| **Site** | **Unique ID** | **Taxon** | **Context** | **dP4** | **P4** | **M1** | **M2** | **M3** | **Stage** | **Age** |
| --- | --- | --- | --- | --- | --- | --- | --- | --- | --- | --- |
| Pre-4th century Magoula Plataniotiki | 475 | Goat | U3202.5 | - | - | - | - | 9G | F | 3-4y |
| Classical Magoula Plataniotiki | 815 | Goat | U2111.7 | 7L | - | - | - | - | B | 2-6m |
| Classical Magoula Plataniotiki | 852 | Goat | U2124.2 | 14L | - | 2A | - | - | C | 6-12m |
| Classical Magoula Plataniotiki | 878 | Goat | U2131.1 | - | 15A | 12A-15A | - | - | G-H-I | 4-10y |
| Classical Magoula Plataniotiki | 442 | Sheep | U3008.2 | - | - | - | 12A | 11G | H | 6-8y |
| Classical Magoula Plataniotiki | 1041 | Sheep | U6581.2 | 14L | - | 6A | - | - | C | 6-12m |
| Classical Magoula Plataniotiki | 159 | Sheep | U6203.1 | - | 9A | 9A | 9A | 5A | E | 2-3y |
| Classical Magoula Plataniotiki | 161 | Sheep | U6203.3 | - | - | 9A | - | 11G | G | 4-6y |
| Classical Magoula Plataniotiki | 229 | Sheep | U6485.1 | - | - | 9A | 8B | 5A | E | 2-3y |
| Classical Magoula Plataniotiki | 516 | Sheep | U3241.12 | - | - | 10B | 9A | 10H | F | 3-4y |
| Classical Magoula Plataniotiki | 1233 | Sheep/goat | U6681.5 | - | - | - | - | 11G (Late) | Η | 6-8y |
| Classical Magoula Plataniotiki | 1204 | Goat | U6628.2 | - | - | - | - | 8G | F | 3-4y |
| Classical Magoula Plataniotiki | 1210 | Sheep/goat | U6630.2 | 16L | - | 7A | - | - | C | 6-12m |
| Hellenistic Magoula Plataniotiki | 750 | Sheep | U2004.5 | - | - | - | - | 10H | F | 3-4y |
| Hellenistic Magoula Plataniotiki | 994 | Sheep/goat | U4442.2 | - | - | - | - | 0 | D | 1-2y |
| Hellenistic Magoula Plataniotiki | 366 | Sheep | U4418.3 | - | - | - | - | 14G | I | 8-10y |
| Hellenistic Magoula Plataniotiki | 1045 | Sheep | U4471.1 | - | - | - | - | 9G | F | 3-4y |
| Hellenistic Magoula Plataniotiki | 1066 | Sheep | U4459.3 | - | - | - | - | 11G | G-H | 4-8y |
| New Halos | - | Sheep/goat | 249.02 | - | - | - | - | 0 | D | 1-2y |
| New Halos | - | Sheep/goat | 224.32 | - | - | - | - | 0 | D | 1-2y |
| New Halos | - | Sheep/goat | 178.-- | - | - | - | - | 0 | D | 1-2y |
| New Halos | - | Sheep | 127.61 | - | - | 12A | 11A | 11G | H | 6-8y |
| New Halos | - | Sheep/goat | 127.61 | - | - | - | - | 7A-9G | E-F | 2-4y |
| New Halos | - | Sheep | 117.03 | - | - | - | - | 10G/11G | F-G-H | 3-8y |
| New Halos | - | Sheep/goat | 224.35 | - | 4A | 9A | - | - | D-E | 1-3y |
| New Halos | - | Sheep/goat | H08-417/18 | - | 4A | - | - | - | D-E | 1-3y |
| New Halos | - | Sheep/goat | H08-404/9 | - | 9A | - | - | - | E-F-G | 2-6y |
| New Halos | - | Sheep | 127.53 | - | 12S | 12A | 9A | - | F-G | 3-6y |
| New Halos | - | Sheep | 150.06 | - | - | - | - | 11G | G-H | 4-8y |
| New Halos | - | Sheep/goat | 127.74 | - | - | - | 9A | 9G | F | 3-4y |
| New Halos | - | Goat | 127.74 | - | 12S | 12A | 9A | - | F-G | 3-6y |
| New Halos | - | Sheep | 127.31 | - | - | - | - | 11G | G-H | 4-8y |
| New Halos | - | Sheep | 127.58 | - | - | - | - | 11G | G-H | 4-8y |
| New Halos | - | Sheep/goat | 231.04 | - | - | - | - | 4A | E | 2-3y |
| Pherae | 1975 | Sheep | E_Chadj | 13L | - | 2A | - | - | C | 6-12m |
| Pherae | 2103 | Sheep | E_Tsoumbekou | 14L | - | 5A | - | - | C | 6-12m |
| Pherae | 1228 | Sheep | N_Tsekou | 14L | - | - | - | - | C | 6-12m |
| Pherae | 1936 | Goat | A_Apostolina | 16L | - | 9A | Erupting | - | C | 6-12m |
| Pherae | 1168 | Sheep | N_Tsekou | 16L | Under dP4 | 9A | 4A | - | D | 1-2y |
| Pherae | 1133 | Sheep | N_Tsekou | - | 3B | 9A | 7A | - | D | 1-2y |
| Pherae | 1170 | Sheep/goat | N_Tsekou | 20L | - | 8A-9A | 1B-4A | - | D | 1-2y |
| Pherae | 1637 | Goat | V_Chadj | - | 3B | 9A | 6A? | - | D | 1-2y |
| Pherae | 2471 | Goat | E_Tsoumbekou | 17L | - | 8A/9A | - | - | D | 1-2y |
| Pherae | 1700 | Sheep/goat | V_Chadj | - | - | - | - | 2A | E | 2-3y |
| Pherae | 1964 | Sheep/goat | E_Chadj | - | - | - | - | 5G-6G | F | 3-4y |
| Pherae | 1325 | Sheep | V_Chadj | - | - | - | - | 10G | F | 3-4y |
| Pherae | 1395 | Sheep | V_Chadj | - | - | - | - | 7G | F | 3-4y |
| Pherae | 1617 | Sheep | V_Chadj | - | - | - | - | 10G | F | 3-4y |
| Pherae | 1673 | Sheep | V_Chadj | - | - | - | - | 9G | F | 3-4y |
| Pherae | 2333 | Sheep | E_Tsoumbekou | - | 8A | 9A | 9A | 7G | F | 3-4y |
| Pherae | 1167 | Sheep | N_Tsekou | - | 14S | 14A | 9A | 11G | G | 4-6y |
| Pherae | 1440 | Sheep | V_Chadj | - | - | - | 9A | 11G | G | 4-6y |
| Pherae | 1645 | Sheep | V_Chadj | - | - | - | 9A | 11G | G | 4-6y |
| Pherae | 1940 | Sheep | A_Apostolina | - | - | 11B | 9A | 11G | G | 4-6y |
| Pherae | 2069 | Sheep | E_Tsoumbekou | - | - | - | 9A | 11G | G | 4-6y |
| Pherae | 1727 | Goat | V_Chadj | - | - | 13A | 9A | 11G | G | 4-6y |
| Pherae | 1169 | Sheep | N_Tsekou | 8L-14L | - | 3A-4A | In chamber | - | B-C | 2-12m |
| Pherae | 1291 | Sheep/goat | V_Chadj | - | - | - | Erupting | In chamber | B-C | 2-12m |
| Pherae | 1478 | Sheep/goat | V_Chadj | 13L | - | Erupting | - | - | B-C | 2-12m |
| Pherae | 1659 | Sheep/goat | V_Chadj | - | - | - | - | Erupting?Unworn? | C-D | 6m-2y |
| Pherae | 1816 | Sheep | A_Apostolina | - | 8A | 9A | 9A | - | E-F | 2-4y |
| Pherae | 1518 | Sheep | V_Chadj | - | 11S | 13A | 9A | In wear | F-G | 3-6y |
| Pherae | 1606 | Sheep | V_Chadj | - | 11S | 12A | - | 11G | F-G | 3-6y |
| Pherae | 1701 | Sheep | V_Chadj | - | In wear | 10A | 9A | In wear | F-G | 3-6y |
| Pherae | 2057 | Sheep | E_Chadj | - | - | 14A | - | - | F-G | 3-6y |
| Pherae | 2307 | Sheep | E_Tsoumbekou | - | - | 15A | 9A | - | F-G | 3-6y |
| Pherae | 1202 | Sheep | N_Tsekou | - | - | - | - | 11G | G-H | 4-8y |
| Pherae | 2490 | Sheep | V_Chadj | - | - | - | - | 11G | G-H | 4-8y |
| Pherae | 1565 | Sheep/goat | V_Chadj | - | - | - | - | 11G? | G-H | 4-8y |
| Pherae | 1678 | Sheep/goat | V_Chadj | - | - | - | 15A | - | H-I | 6-10y |
| Pherae | 1974 | Sheep | E_Chadj | - | - | - | 15A | - | H-I | 6-10y |
| Pherae | 2005 | Goat | E_Chadj | - | - | - | 8A/9A | - | E-F-G | 2-6y |
| Pherae | 1746 | Sheep | V_Chadj | - | 14S | - | - | - | F-G-H | 3-8y |

**S3 Table.** **Sheep/goat ageing data in Magoula Plataniotiki, New Halos, and Pherae based on mandibular tooth wear and eruption after** **Payne [1,2]**. Deciduous fourth premolar= dP4. Permanent fourth premolar= P4, first molar= M1, second molar= M2, third molar= M3.

# **References**

1. Payne S. Kill-off Patterns in Sheep and Goats: The Mandibles from Aşvan Kale. Anatol Stud [Internet]. 1973;23:281–303. Available from: http://www.jstor.org/stable/3642547

2. Payne S. Reference codes for wear states in the mandibular cheek teeth of sheep and goats. J Archaeol Sci. 1987;14(6):609–14.
