# Supplementary material for: Where did the herds go? Combining zooarchaeological and isotopic data to examine animal management in ancient Thessaly (Greece)
Source: PLoS One. 2024 Oct 22;19(10):e0299788. doi: 10.1371/journal.pone.0299788 (PMC11495569; doi:10.1371/journal.pone.0299788)
Supplement: S4 Table — (*) The three fusing distal tibiae and one fusing distal metacarpal are listed under the fused category. (DOCX) [file pone.0299788.s010.docx]

Supporting Information- Tables

| **Age of fusion** | **Element** | **Classical Magoula Plataniotiki** | | **Hellenistic Magoula Plataniotiki** | | **Pherae** | |
| --- | --- | --- | --- | --- | --- | --- | --- |
|  |  | **Unfused** | **Fused** | **Unfused** | **Fused** | **Unfused** | **Fused** |
| 7-8m | Pelvis acetabulum | 1 | 1 | 1 | 1 | 0 | 2 |
| 7-8m | Scapula distal | 0 | 2 | 0 | 1 | 1 | 4 |
| 7-8m | Radius proximal | 1 | 0 | 0 | 1 | 1 | 2 |
| 8-18m | 2nd Phalanx | 2 | 2 | 0 | 1 | 0 | 6 |
| 8-18m | Humerus distal | 0 | 2 | 0 | 0 | 3 | 8 |
| 18-24m | 1st Phalanx | 2 | 3 | 1 | 1 | 5 | 7 |
| 18-24m | Tibia distal | 0 | 1 | 2 | 0 | 7 | 10* |
| 24-36m | Metatarsal distal | 1 | 1 | 0 | 0 | 9 | 3 |
| 24-36m | Metacarpal distal | 2 | 2 | 1 | 0 | 5 | 4* |
| 24-36m | Metapodial distal | 1 | 2 | 2 | 1 | 4 | 1 |
| 36-48m | Calcaneus proximal | 1 | 0 | 1 | 0 | 10 | 1 |
| 36-48m | Femur proximal | 1 | 0 | 1 | 0 | 0 | 0 |
| 48-60m | Radius distal | 1 | 0 | 0 | 0 | 5 | 0 |
| 48-60m | Ulna proximal | 0 | 0 | 1 | 0 | 5 | 0 |
| 48-60m | Femur distal | 1 | 0 | 0 | 0 | 2 | 0 |
| 48-60m | Tibia proximal | 1 | 0 | 0 | 0 | 6 | 2 |
| 48-60m | Humerus proximal | 2 | 0 | 1 | 0 | 3 | 0 |

**S4 Table.** **Pig ageing data of Classical Magoula Plataniotiki, Hellenistic Magoula Plataniotiki, and Pherae based on epiphyseal fusion after Zeder Lemoine and Payne** [1]**.** (*) The three fusing distal tibiae and one fusing distal metacarpal are listed under the fused category.

# **References**

1. Zeder MA, Lemoine X, Payne S. A new system for computing long-bone fusion age profiles in Sus scrofa. J Archaeol Sci [Internet]. 2015;55:135–50. Available from: https://doi.org/10.1016/j.jas.2014.12.017
