# Supplementary material for: Where did the herds go? Combining zooarchaeological and isotopic data to examine animal management in ancient Thessaly (Greece)
Source: PLoS One. 2024 Oct 22;19(10):e0299788. doi: 10.1371/journal.pone.0299788 (PMC11495569; doi:10.1371/journal.pone.0299788)
Supplement: S5 Table — (*) The single fusing distal tibia is listed under the fused category. Loose epiphyses are excluded. (DOCX) [file pone.0299788.s011.docx]

Supporting Information- Tables

| **Age of fusion** | **Element** | **Classical Magoula Plataniotiki** | | **Hellenistic Magoula Plataniotiki** | | **Pherae** | |
| --- | --- | --- | --- | --- | --- | --- | --- |
|  |  | **Unfused** | **Fused** | **Unfused** | **Fused** | **Unfused** | **Fused** |
| 6-10m | Pelvis acetabulum | 0 | 0 | 0 | 0 | 0 | 1 |
| 7-10m | Scapula proximal | 0 | 0 | 0 | 0 | 0 | 0 |
| 12-18m | Humerus distal | 1 | 2 | 0 | 1 | 0 | 2 |
| 12-18m | Rarius proximal | 0 | 1 | 0 | 3 | 0 | 2 |
| 18-24m | 1st Phalanx | 0 | 5 | 0 | 10 | 0 | 13 |
| 18-24m | 2nd Phalanx | 0 | 5 | 0 | 5 | 0 | 10 |
| 24-30m | Tibia distal | 0 | 0 | 1 | 2 | 2 | 5* |
| 24-36m | Metacarpal distal | 0 | 1 | 0 | 1 | 0 | 0 |
| 24-36m | Metatarsal distal | 0 | 0 | 0 | 2 | 0 | 3 |
| 24-36m | Metapodial distal | 0 | 0 | 0 | 0 | 0 | 1 |
| 36-42m | Calcaneus proximal | 0 | 1 | 3 | 0 | 0 | 3 |
| 42-48m | Humerus proximal | 0 | 1 | 0 | 0 | 0 | 1 |
| 42-48m | Radius distal | 1 | 0 | 1 | 1 | 1 | 3 |
| 42-48m | Ulna proximal | 0 | 0 | 0 | 0 | 0 | 0 |
| 42m | Femur proximal | 1 | 0 | 1 | 2 | 0 | 0 |
| 42-48m | Femur distal | 0 | 0 | 0 | 1 | 1 | 1 |
| 42-48m | Tibia proximal | 0 | 0 | 0 | 1 | 0 | 0 |

**S5 Table.** **Cattle ageing data of Classical Magoula Plataniotiki, Hellenistic Magoula Plataniotiki, and Pherae based on epiphyseal fusion after Reitz and Wing [1].**
(*) The single fusing distal tibia is listed under the fused category. Loose epiphyses are excluded.

# **References**

1. Reitz EJ, Wing ES. Zooarchaeology. 2nd ed. Acta Universitatis Agriculturae et Silviculturae Mendelianae Brunensis. Cambridge: Cambridge University Press; 2008.
