# Supplementary material for: Where did the herds go? Combining zooarchaeological and isotopic data to examine animal management in ancient Thessaly (Greece)
Source: PLoS One. 2024 Oct 22;19(10):e0299788. doi: 10.1371/journal.pone.0299788 (PMC11495569; doi:10.1371/journal.pone.0299788)
Supplement: S6 Table — The results were compared to a database of published refences markers [6–8]. The annotated peaks represent the markers used to make the identifications, specifically marker 3033.4 for sheep and 3093.4 for goat. (DOCX) [file pone.0299788.s012.docx]

Supporting Information- Tables

|  |  |  | **ZooMS peptide markers** | | | | | | | | | | | |
| --- | --- | --- | --- | --- | --- | --- | --- | --- | --- | --- | --- | --- | --- | --- |
| **ZooMS ID** | **Sample ID** | **Results** | **ɑ1 508** | **ɑ2 978** | **ɑ2 978 (+Hyp)** | **ɑ2 484** | **ɑ2 502** | **ɑ2 292** | **ɑ2 793** | **ɑ2 454** | **ɑ1 586** | **ɑ1 586 (+Hyp)** | **ɑ2 757** | **ɑ2 757 (+Hyp)** |
| ZooMS_00190_1 | PH11.00 | Sheep | 1105.6 |  |  | 1427.7 | 1580.8 | 1648.8 | 2131.1 | 2792.4 | 2883.4 | 2899.4 | 3017.4 | 3033.4 |
| ZooMS_00190_2 | PH10.0 | Sheep | 1105.6 |  | 1196.6 | 1427.7 | 1580.8 | 1648.8 | 2131.1 | 2792.4 | 2883.4 | 2899.4 | 3017.4 | 3033.4 |
| ZooMS_00190_6 | NH3.0 | Goat | 1105.6 |  |  | 1427.7 | 1580.8 | 1648.8 | 2131.1 | 2792.4 | 2883.4 | 2899.4 |  | 3093.4 |
| ZooMS_00190_7 | NH2.0 | Sheep | 1105.6 |  |  | 1427.7 | 1580.8 | 1648.8 | 2131.1 | 2792.4 | 2883.4 | 2899.4 | 3017.4 | 3033.4 |

**
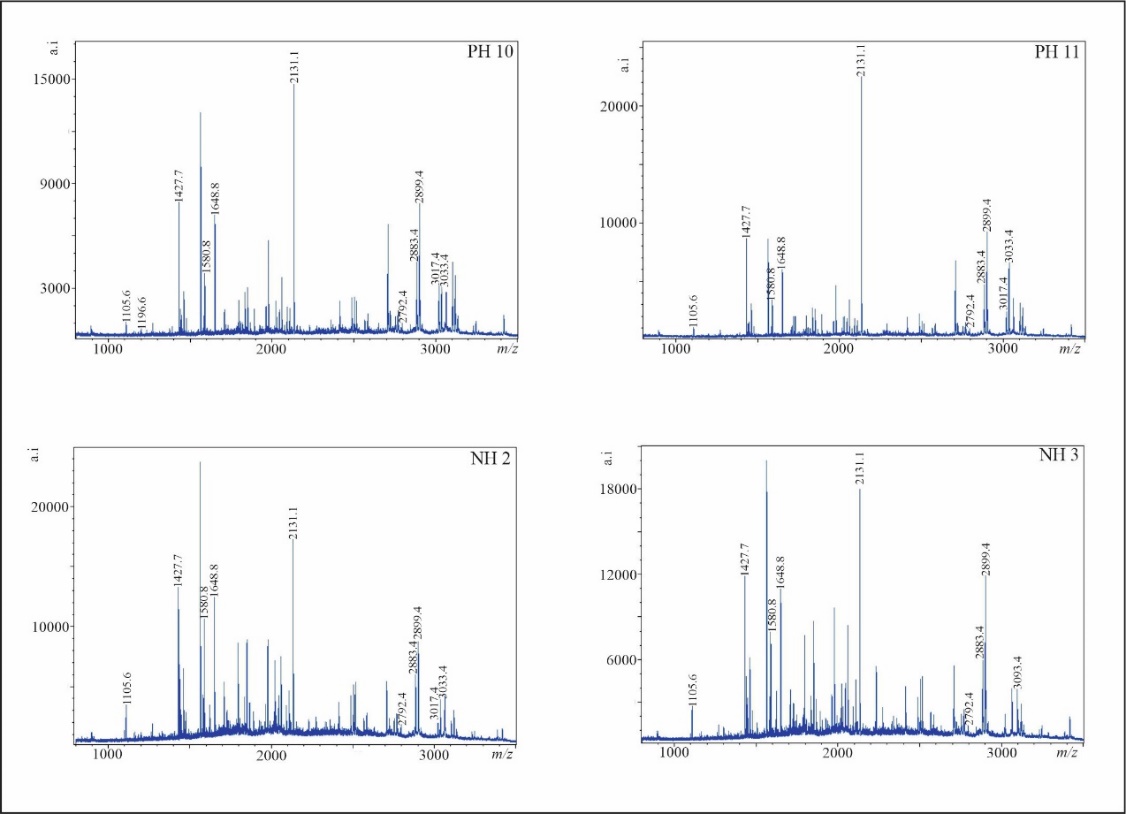
**

**S6 Table. ZooMS collagen peptide mass fingerprint spectra of NH2 (00190-7), NH3 (00190-6), PH10 (00190-2), and PH11 (00190-1).** The results were compared to a database of published refences markers [1–3]. The annotated peaks represent the markers used to make the identifications, specifically marker 3033.4 for sheep and 3093.4 for goat.

# **References**

1. Buckley M, Collins M, Thomas-Oates J, Wilson JC. Species identification by analysis of bone collagen using matrix-assisted laser desorption/ionisation time-of-flight mass spectrometry. Rapid Commun Mass Spectrom [Internet]. 2009;23:3843–54. Available from: https://doi.org/10.1002/rcm.4316

2. Buckley M, Kansa SW, Howard S, Campbell S, Thomas-Oates J, Collins M. Distinguishing between archaeological sheep and goat bones using a single collagen peptide. J Archaeol Sci [Internet]. 2010;37(1):13–20. Available from: http://dx.doi.org/10.1016/j.jas.2009.08.020

3. Welker F, Hajdinjak M, Talamo S, Jaouen K, Dannemann M, David F, et al. Palaeoproteomic Evidence Identifies Archaic Hominins Associated with the Châtelperronian at the Grotte Du Renne. Proceedings of the National Academy of Sciences of the United States of America; 2016.
