# Supplementary material for: Where did the herds go? Combining zooarchaeological and isotopic data to examine animal management in ancient Thessaly (Greece)
Source: PLoS One. 2024 Oct 22;19(10):e0299788. doi: 10.1371/journal.pone.0299788 (PMC11495569; doi:10.1371/journal.pone.0299788)
Supplement: S7 Table — 2σ = 2 standard deviation. (DOCX) [file pone.0299788.s013.docx]

Supporting Information- Tables

| **Site** | **Context** | **Date** | **Taxon** | **Element** | **Sample ID** | **δ^13^C (‰ VPDB)** | **δ^18^O (‰ VPDB)** | **^87^/^86^Sr brack corr** | **2σ** | **Distance from ERJ (mm)** |
| --- | --- | --- | --- | --- | --- | --- | --- | --- | --- | --- |
| Classical Magoula Plataniotiki | U6485.1 | Early-Middle 4^th^ century BCE | Sheep | LM3.2 | HA 1.1 | -11,13 | -3,70 | 0,708816 | 0,000012 | 4,0 |
| Classical Magoula Plataniotiki | U6485.1 | Early-Middle 4^th^ century BCE | Sheep | LM3.2 | HA 1.2 | -11,86 | -3,61 | 0,708804 | 0,000012 | 6,0 |
| Classical Magoula Plataniotiki | U6485.1 | Early-Middle 4^th^ century BCE | Sheep | LM3.2 | HA 1.3 | -11,36 | -3,16 | - | - | 9,0 |
| Classical Magoula Plataniotiki | U6485.1 | Early-Middle 4^th^ century BCE | Sheep | LM3.2 | HA 1.4 | -10,50 | -2,78 | 0,708808 | 0,000012 | 11,0 |
| Classical Magoula Plataniotiki | U6485.1 | Early-Middle 4^th^ century BCE | Sheep | LM3.2 | HA 1.5 | -10,12 | -2,65 | - | - | 13,5 |
| Classical Magoula Plataniotiki | U6485.1 | Early-Middle 4^th^ century BCE | Sheep | LM3.2 | HA 1.6 | -9,82 | -2,66 | - | - | 16,0 |
| Classical Magoula Plataniotiki | U6485.1 | Early-Middle 4^th^ century BCE | Sheep | LM3.2 | HA 1.7 | - | - | 0,708857 | 0,000012 | 18,0 |
| Classical Magoula Plataniotiki | U6485.1 | Early-Middle 4^th^ century BCE | Sheep | LM3.2 | HA 1.8 | -10,02 | -2,70 | - | - | 21,0 |
| Classical Magoula Plataniotiki | U6485.1 | Early-Middle 4^th^ century BCE | Sheep | LM3.2 | HA 1.9 | -9,98 | -1,19 | - | - | 23,0 |
| Classical Magoula Plataniotiki | U6485.1 | Early-Middle 4^th^ century BCE | Sheep | LM3.2 | HA 1.10 | - | - | 0,708890 | 0,000012 | 26,0 |
| Classical Magoula Plataniotiki | U6485.1 | Early-Middle 4^th^ century BCE | Sheep | LM3.2 | HA 1.11 | -10,82 | -0,42 | - | - | 28,0 |
| Classical Magoula Plataniotiki | U6203.1 | Early-Middle 4^th^ century BCE | Sheep | LM2.2 | HA 2.1 | -11,91 | -4,85 | - |  | 2,6 |
| Classical Magoula Plataniotiki | U6203.1 | Early-Middle 4^th^ century BCE | Sheep | LM2.2 | HA 2.2 | -10,92 | -3,60 | 0,708880 | 0,000012 | 5,0 |
| Classical Magoula Plataniotiki | U6203.1 | Early-Middle 4^th^ century BCE | Sheep | LM2.2 | HA 2.3 | -10,42 | -2,65 | 0,708884 | 0,000012 | 6,9 |
| Classical Magoula Plataniotiki | U6203.1 | Early-Middle 4^th^ century BCE | Sheep | LM2.2 | HA 2.4 | -10,54 | -1,71 | - | - | 8,8 |
| Classical Magoula Plataniotiki | U6203.1 | Early-Middle 4^th^ century BCE | Sheep | LM2.2 | HA 2.5 | -11,15 | -1,23 | 0,709092 | 0,000012 | 10,5 |
| Classical Magoula Plataniotiki | U6203.1 | Early-Middle 4^th^ century BCE | Sheep | LM2.2 | HA 2.6 | -12,04 | -1,64 | - | - | 12,7 |
| Classical Magoula Plataniotiki | U6203.1 | Early-Middle 4^th^ century BCE | Sheep | LM2.2 | HA 2.7 | -12,87 | -3,34 | 0,709234 | 0,000012 | 15,3 |
| Classical Magoula Plataniotiki | U6203.1 | Early-Middle 4^th^ century BCE | Sheep | LM2.2 | HA 2.8 | -13,58 | -4,29 | - | - | 18,1 |
| Classical Magoula Plataniotiki | U6203.1 | Early-Middle 4^th^ century BCE | Sheep | LM2.2 | HA 2.9 | -13,46 | -5,39 | - | - | 20,9 |
| Classical Magoula Plataniotiki | U6203.1 | Early-Middle 4^th^ century BCE | Sheep | LM2.2 | HA 2.10 | -13,26 | -7,34 | 0,709215 | 0,000012 | 23,8 |
| Classical Magoula Plataniotiki | U6203.3 | Early-Middle 4^th^ century BCE | Sheep | LM3.2 | HA 3.1 | -11,21 | -2,49 | 0,709075 | 0,000012 | 3,6 |
| Classical Magoula Plataniotiki | U6203.3 | Early-Middle 4^th^ century BCE | Sheep | LM3.2 | HA 3.2 | -11,56 | -1,22 | - | - | 6,2 |
| Classical Magoula Plataniotiki | U6203.3 | Early-Middle 4^th^ century BCE | Sheep | LM3.2 | HA 3.3 | -13,31 | -3,36 | 0,708930 | 0,000012 | 8,9 |
| Classical Magoula Plataniotiki | U6203.3 | Early-Middle 4^th^ century BCE | Sheep | LM3.2 | HA 3.4 | -13,65 | -4,21 | - | - | 11,4 |
| Classical Magoula Plataniotiki | U6203.3 | Early-Middle 4^th^ century BCE | Sheep | LM3.2 | HA 3.5 | -13,46 | -4,54 | 0,708906 | 0,000012 | 13,9 |
| Classical Magoula Plataniotiki | U6203.3 | Early-Middle 4^th^ century BCE | Sheep | LM3.2 | HA 3.6 | -12,74 | -4,25 | - | - | 16,8 |
| Classical Magoula Plataniotiki | U6203.3 | Early-Middle 4^th^ century BCE | Sheep | LM3.2 | HA 3.7 | -12,18 | -4,23 | 0,708939 | 0,000012 | 19,0 |
| Classical Magoula Plataniotiki | U6203.3 | Early-Middle 4^th^ century BCE | Sheep | LM3.2 | HA 3.8 | -11,72 | -4,19 | - | - | 21,1 |
| Classical Magoula Plataniotiki | U6203.3 | Early-Middle 4^th^ century BCE | Sheep | LM3.2 | HA 3.9 | -10,94 | -3,76 | 0,708978 | 0,000012 | 23,2 |
| Hellenistic Magoula Plataniotiki | U4471.1 | 3^rd^ century BCE | Sheep | LM3.2 | HA 4.1 | -11,35 | -4,46 | 0,709416 | 0,000012 | 2,7 |
| Hellenistic Magoula Plataniotiki | U4471.1 | 3^rd^ century BCE | Sheep | LM3.2 | HA 4.2 | -12,10 | -4,14 | - | - | 4,6 |
| Hellenistic Magoula Plataniotiki | U4471.1 | 3^rd^ century BCE | Sheep | LM3.2 | HA 4.3 | -12,06 | -3,42 | - | - | 7,0 |
| Hellenistic Magoula Plataniotiki | U4471.1 | 3^rd^ century BCE | Sheep | LM3.2 | HA 4.4 | -12,26 | -3,00 | 0,709450 | 0,000012 | 9,3 |
| Hellenistic Magoula Plataniotiki | U4471.1 | 3^rd^ century BCE | Sheep | LM3.2 | HA 4.5 | -12,50 | -3,15 | - | - | 11,6 |
| Hellenistic Magoula Plataniotiki | U4471.1 | 3^rd^ century BCE | Sheep | LM3.2 | HA 4.6 | -13,05 | -4,63 | - | - | 13,4 |
| Hellenistic Magoula Plataniotiki | U4471.1 | 3^rd^ century BCE | Sheep | LM3.2 | HA 4.7 | -13,25 | -5,75 | 0,709187 | 0,000012 | 15,7 |
| Hellenistic Magoula Plataniotiki | U4471.1 | 3^rd^ century BCE | Sheep | LM3.2 | HA 4.8 | -13,20 | -6,85 | - | - | 18,1 |
| Hellenistic Magoula Plataniotiki | U4471.1 | 3^rd^ century BCE | Sheep | LM3.2 | HA 4.9 | -12,92 | -7,47 | - | - | 20,9 |
| Hellenistic Magoula Plataniotiki | U4471.1 | 3^rd^ century BCE | Sheep | LM3.2 | HA 4.10 | -12,43 | -7,74 | 0,709258 | 0,000012 | 22,9 |
| Hellenistic Magoula Plataniotiki | U4471.1 | 3^rd^ century BCE | Sheep | LM3.2 | HA 4.11 | -12,02 | -7,26 | - | - | 25,2 |
| Hellenistic Magoula Plataniotiki | U4471.1 | 3^rd^ century BCE | Sheep | LM3.2 | HA 4.12 | -11,48 | -7,01 | - | - | 27,7 |
| Hellenistic Magoula Plataniotiki | U4471.1 | 3^rd^ century BCE | Sheep | LM3.2 | HA 4.13 | -10,89 | -6,79 | 0,709253 | 0,000012 | 30,3 |
| Pre-4^th^ ce BCE Magoula Plataniotiki | U3202.5 | 8^th^-5^th^ century BCE | Goat | LM3.2 | HA 5.1 | -12,24 | -3,56 | 0,708602 | 0,000012 | 1,7 |
| Pre-4^th^ ce BCE Magoula Plataniotiki | U3202.5 | 8^th^-5^th^ century BCE | Goat | LM3.2 | HA 5.2 | -12,53 | -2,95 | - | - | 4,5 |
| Pre-4^th^ ce BCE Magoula Plataniotiki | U3202.5 | 8^th^-5^th^ century BCE | Goat | LM3.2 | HA 5.3 | -12,17 | -2,13 | 0,708750 | 0,000014 | 6,9 |
| Pre-4^th^ ce BCE Magoula Plataniotiki | U3202.5 | 8^th^-5^th^ century BCE | Goat | LM3.2 | HA 5.4 | -12,11 | -2,02 | - | - | 9,3 |
| Pre-4^th^ ce BCE Magoula Plataniotiki | U3202.5 | 8^th^-5^th^ century BCE | Goat | LM3.2 | HA 5.5 | -11,86 | -1,46 | 0,709524 | 0,000017 | 11,3 |
| Pre-4^th^ ce BCE Magoula Plataniotiki | U3202.5 | 8^th^-5^th^ century BCE | Goat | LM3.2 | HA 5.6 | -11,89 | -2,68 | 0,709667 | 0,000022 | 13,9 |
| Pre-4^th^ ce BCE Magoula Plataniotiki | U3202.5 | 8^th^-5^th^ century BCE | Goat | LM3.2 | HA 5.7 | -12,28 | -3,30 | - | - | 16,6 |
| Pre-4^th^ ce BCE Magoula Plataniotiki | U3202.5 | 8^th^-5^th^ century BCE | Goat | LM3.2 | HA 5.8 | -12,42 | -3,95 | 0,709772 | 0,000023 | 18,7 |
| Hellenistic Magoula Plataniotiki | U2004.5 | 3^rd^ century BCE | Sheep | LM3.2 | HA 6.1 | -9,79 | -3,61 | 0,709080 | 0,000012 | 6,8 |
| Hellenistic Magoula Plataniotiki | U2004.5 | 3^rd^ century BCE | Sheep | LM3.2 | HA 6.2 | -8,93 | -2,44 | - | - | 9,0 |
| Hellenistic Magoula Plataniotiki | U2004.5 | 3^rd^ century BCE | Sheep | LM3.2 | HA 6.3 | -9,47 | -2,81 | - | - | 11,0 |
| Hellenistic Magoula Plataniotiki | U2004.5 | 3^rd^ century BCE | Sheep | LM3.2 | HA 6.4 | -11,11 | -3,70 | 0,709083 | 0,000012 | 13,0 |
| Hellenistic Magoula Plataniotiki | U2004.5 | 3^rd^ century BCE | Sheep | LM3.2 | HA 6.5 | -12,29 | -4,19 | - | - | 15,0 |
| Hellenistic Magoula Plataniotiki | U2004.5 | 3^rd^ century BCE | Sheep | LM3.2 | HA 6.6 | -13,40 | -5,11 | - | - | 17,0 |
| Hellenistic Magoula Plataniotiki | U2004.5 | 3^rd^ century BCE | Sheep | LM3.2 | HA 6.7 | -13,72 | -5,38 | 0,709136 | 0,000012 | 19,5 |
| Hellenistic Magoula Plataniotiki | U2004.5 | 3^rd^ century BCE | Sheep | LM3.2 | HA 6.8 | -13,92 | -5,68 | - | - | 22,0 |
| Hellenistic Magoula Plataniotiki | U2004.5 | 3^rd^ century BCE | Sheep | LM3.2 | HA 6.9 | - | - | 0,709137 | 0,000012 | 24,5 |
| Hellenistic Magoula Plataniotiki | U2004.5 | 3^rd^ century BCE | Sheep | LM3.2 | HA 6.10 | -12,63 | -4,72 | - | - | 27,0 |
| Hellenistic Magoula Plataniotiki | U2004.5 | 3^rd^ century BCE | Sheep | LM3.2 | HA 6.11 | -11,39 | -4,26 | 0,709115 | 0,000012 | 30,0 |
| Pre-4^th^ ce BCE Magoula Plataniotiki | U3117.1 | 12^th^-8^th^ century BCE | Cattle | LM3.2 | HA 9.1 | -10,61 | -5,05 | 0,712147 | 0,000012 | 3,0 |
| Pre-4^th^ ce BCE Magoula Plataniotiki | U3117.1 | 12^th^-8^th^ century BCE | Cattle | LM3.2 | HA 9.2 | -10,57 | -5,51 | 0,712210 | 0,000012 | 5,2 |
| Pre-4^th^ ce BCE Magoula Plataniotiki | U3117.1 | 12^th^-8^th^ century BCE | Cattle | LM3.2 | HA 9.3 | -10,30 | -4,61 | 0,712208 | 0,000012 | 7,7 |
| Pre-4^th^ ce BCE Magoula Plataniotiki | U3117.1 | 12^th^-8^th^ century BCE | Cattle | LM3.2 | HA 9.3 rep | -10,38 | -4,64 | - | - | 7,7 |
| Pre-4^th^ ce BCE Magoula Plataniotiki | U3117.1 | 12^th^-8^th^ century BCE | Cattle | LM3.2 | HA 9.4 | -10,28 | -4,70 | 0,712257 | 0,000012 | 10,3 |
| Hellenistic Magoula Plataniotiki | U4457.2 | 3^rd^ century BCE | Cattle | LM2.2 | HA 10.1 | -11,90 | -5,31 | - | - | 5,5 |
| Hellenistic Magoula Plataniotiki | U4457.2 | 3^rd^ century BCE | Cattle | LM2.2 | HA 10.2 | -11,74 | -5,01 | 0,708691 | 0,000012 | 8,4 |
| Hellenistic Magoula Plataniotiki | U4457.2 | 3^rd^ century BCE | Cattle | LM2.2 | HA 10.3 | -11,42 | -3,90 | - | - | 10,7 |
| Hellenistic Magoula Plataniotiki | U4457.2 | 3^rd^ century BCE | Cattle | LM2.2 | HA 10.4 | -11,63 | -4,33 | - | - | 13,8 |
| Hellenistic Magoula Plataniotiki | U4457.2 | 3^rd^ century BCE | Cattle | LM2.2 | HA 10.4 rep | -11,65 | -4,21 | - | - | 13,8 |
| Hellenistic Magoula Plataniotiki | U4457.2 | 3^rd^ century BCE | Cattle | LM2.2 | HA 10.5 | -11,34 | -2,51 | - | - | 16,8 |
| Hellenistic Magoula Plataniotiki | U4457.2 | 3^rd^ century BCE | Cattle | LM2.2 | HA 10.6 | -11,25 | -2,78 | 0,708622 | 0,000012 | 20,1 |
| Hellenistic Magoula Plataniotiki | U4457.2 | 3^rd^ century BCE | Cattle | LM2.2 | HA 10.7 | -11,55 | -2,95 | - | - | 23,2 |
| Hellenistic Magoula Plataniotiki | U4457.2 | 3^rd^ century BCE | Cattle | LM2.2 | HA 10.8 | -11,64 | -2,87 | 0,708657 | 0,000012 | 26,6 |
| Hellenistic Magoula Plataniotiki | U4457.2 | 3^rd^ century BCE | Cattle | LM2.2 | HA 10.9 | -12,27 | -3,28 | - | - | 29,8 |
| Hellenistic Magoula Plataniotiki | U4457.2 | 3^rd^ century BCE | Cattle | LM2.2 | HA 10.9 rep | -12,34 | -3,19 | - | - | 29,8 |
| Hellenistic Magoula Plataniotiki | U4457.2 | 3^rd^ century BCE | Cattle | LM2.2 | HA 10.10 | -12,17 | -2,76 | 0,708672 | 0,000012 | 33,2 |
| Hellenistic Magoula Plataniotiki | U4457.2 | 3^rd^ century BCE | Cattle | LM2.2 | HA 10.11 | -12,28 | -2,74 | - | - | 36,9 |
| Hellenistic Magoula Plataniotiki | U4457.2 | 3^rd^ century BCE | Cattle | LM2.2 | HA 10.12 | -12,85 | -3,64 | 0,708738 | 0,000012 | 40,6 |
| Hellenistic New Halos | House of Amphorae | Early-Middle 3^rd^ century BCE | Sheep | LM3.2 | NH 1.1 | -7,18 | -2,58 | 0,708636 | 0,000012 | 2,8 |
| Hellenistic New Halos | House of Amphorae | Early-Middle 3^rd^ century BCE | Sheep | LM3.2 | NH 1.2 | -8,31 | -2,56 | - | - | 5,9 |
| Hellenistic New Halos | House of Amphorae | Early-Middle 3^rd^ century BCE | Sheep | LM3.2 | NH 1.3 | -10,93 | -4,49 | 0,708620 | 0,000012 | 8,5 |
| Hellenistic New Halos | House of Amphorae | Early-Middle 3^rd^ century BCE | Sheep | LM3.2 | NH 1.4 | -12,10 | -6,52 | 0,708646 | 0,000012 | 11,6 |
| Hellenistic New Halos | House of Amphorae | Early-Middle 3^rd^ century BCE | Sheep | LM3.2 | NH 1.5 | -11,50 | -6,51 | - | - | 14,4 |
| Hellenistic New Halos | House of Amphorae | Early-Middle 3^rd^ century BCE | Sheep | LM3.2 | NH 1.6 | -9,00 | -4,81 | 0,708697 | 0,000012 | 17,2 |
| Hellenistic New Halos | House of Amphorae | Early-Middle 3^rd^ century BCE | Sheep | LM3.2 | NH 1.7 | -7,56 | -3,90 | 0,708719 | 0,000012 | 19,7 |
| Hellenistic New Halos | House of Amphorae | Early-Middle 3^rd^ century BCE | Sheep | LM3.1 | NH2.1 | -11,46 | -0,34 | 0,709217 | 0,000020 | 2,4 |
| Hellenistic New Halos | House of Amphorae | Early-Middle 3^rd^ century BCE | Sheep | LM3.1 | NH2.2 | -11,92 | -1,45 | - | - | 4,6 |
| Hellenistic New Halos | House of Amphorae | Early-Middle 3^rd^ century BCE | Sheep | LM3.1 | NH2.3 | -11,75 | -2,05 | - | - | 6,3 |
| Hellenistic New Halos | House of Amphorae | Early-Middle 3^rd^ century BCE | Sheep | LM3.1 | NH2.4 | -11,66 | -2,22 | 0,709087 | 0,000019 | 8,3 |
| Hellenistic New Halos | House of Amphorae | Early-Middle 3^rd^ century BCE | Sheep | LM3.1 | NH2.5 | -11,28 | -2,07 | - | - | 10,5 |
| Hellenistic New Halos | House of Amphorae | Early-Middle 3^rd^ century BCE | Sheep | LM3.1 | NH2.6 | -10,95 | -1,58 | 0,709155 | 0,000020 | 12,4 |
| Hellenistic New Halos | House of Amphorae | Early-Middle 3^rd^ century BCE | Sheep | LM3.1 | NH2.7 | -10,35 | -1,89 | - | - | 14,1 |
| Hellenistic New Halos | House of Amphorae | Early-Middle 3^rd^ century BCE | Sheep | LM3.1 | NH2.8 | -10,67 | -2,68 | 0,709227 | 0,000020 | 16,5 |
| Hellenistic New Halos | House of Amphorae | Early-Middle 3^rd^ century BCE | Sheep | LM3.1 | NH2.9 | -11,08 | -2,95 | - | - | 19,0 |
| Hellenistic New Halos | House of Amphorae | Early-Middle 3^rd^ century BCE | Sheep | LM3.1 | NH2.10 | -11,33 | -3,27 | 0,709368 | 0,000019 | 21,1 |
| Hellenistic New Halos | House of Amphorae | Early-Middle 3^rd^ century BCE | Goat | LM2.2 | NH3.1 | -12,35 | -1,46 | 0,709674 | 0,000019 | 2,1 |
| Hellenistic New Halos | House of Amphorae | Early-Middle 3^rd^ century BCE | Goat | LM2.2 | NH3.2 | -12,77 | -2,86 | - | - | 4,7 |
| Hellenistic New Halos | House of Amphorae | Early-Middle 3^rd^ century BCE | Goat | LM2.2 | NH3.3 | -13,09 | -3,75 | 0,709387 | 0,000025 | 7,1 |
| Hellenistic New Halos | House of Amphorae | Early-Middle 3^rd^ century BCE | Goat | LM2.2 | NH3.4 | -13,12 | -3,72 | 0,709198 | 0,000020 | 9,2 |
| Hellenistic New Halos | House of Amphorae | Early-Middle 3^rd^ century BCE | Goat | LM2.2 | NH3.5 | -13,10 | -3,17 | 0,709138 | 0,000020 | 11,8 |
| Hellenistic New Halos | House of Amphorae | Early-Middle 3^rd^ century BCE | Goat | LM2.2 | NH3.6 | -12,79 | -2,69 | - | - | 14,0 |
| Hellenistic New Halos | House of Amphorae | Early-Middle 3^rd^ century BCE | Goat | LM2.2 | NH3.7 | -13,22 | -2,90 | 0,709130 | 0,000025 | 15,8 |
| Hellenistic New Halos | House of Amphorae | Early-Middle 3^rd^ century BCE | Sheep | LM2.2 | NH4.1 | -11,22 | -1,37 | 0,709282 | 0,000019 | 2,0 |
| Hellenistic New Halos | House of Amphorae | Early-Middle 3^rd^ century BCE | Sheep | LM2.2 | NH4.2 | -11,03 | -0,45 | - | - | 4,2 |
| Hellenistic New Halos | House of Amphorae | Early-Middle 3^rd^ century BCE | Sheep | LM2.2 | NH4.3 | -12,13 | -1,73 | 0,709050 | 0,000017 | 6,6 |
| Hellenistic New Halos | House of Amphorae | Early-Middle 3^rd^ century BCE | Sheep | LM2.2 | NH4.4 | -12,37 | -1,92 | 0,709009 | 0,000017 | 8,8 |
| Hellenistic New Halos | House of Amphorae | Early-Middle 3^rd^ century BCE | Sheep | LM2.2 | NH4.5 | -12,84 | -4,58 | 0,709003 | 0,000017 | 11,0 |
| Hellenistic New Halos | House of Amphorae | Early-Middle 3^rd^ century BCE | Sheep | LM2.2 | NH4.6 | -12,25 | -4,40 | - | - | 13,7 |
| Hellenistic New Halos | House of Amphorae | Early-Middle 3^rd^ century BCE | Sheep | LM2.2 | NH4.7 | -12,01 | -5,19 | 0,709078 | 0,000017 | 15,7 |
| Hellenistic Pherae | V. Chadjitheodorou | 2^nd^-1^st^ century BCE | Cattle | LM3.2 | PH1.1 | -10,95 | -3,51 | 0,708355 | 0,000019 | 2,4 |
| Hellenistic Pherae | V. Chadjitheodorou | 2^nd^-1^st^ century BCE | Cattle | LM3.2 | PH1.2 | -11,02 | -2,03 | - | - | 4,9 |
| Hellenistic Pherae | V. Chadjitheodorou | 2^nd^-1^st^ century BCE | Cattle | LM3.2 | PH1.3 | -10,81 | -2,83 | 0,708342 | 0,000019 | 7,2 |
| Hellenistic Pherae | V. Chadjitheodorou | 2^nd^-1^st^ century BCE | Cattle | LM3.2 | PH1.4 | -10,50 | -2,77 | - | - | 9,6 |
| Hellenistic Pherae | V. Chadjitheodorou | 2^nd^-1^st^ century BCE | Cattle | LM3.2 | PH1.5 | - | - | - | - | 11,9 |
| Hellenistic Pherae | V. Chadjitheodorou | 2^nd^-1^st^ century BCE | Cattle | LM3.2 | PH1.6 | -11,18 | -4,03 | 0,708342 | 0,000019 | 14,2 |
| Hellenistic Pherae | V. Chadjitheodorou | 2^nd^-1^st^ century BCE | Cattle | LM3.2 | PH1.7 | -10,42 | -2,53 | - | - | 16.4 |
| Hellenistic Pherae | V. Chadjitheodorou | 2^nd^-1^st^ century BCE | Cattle | LM3.2 | PH1.8 | -10,21 | -2,50 | 0,708345 | 0,000019 | 18,0 |
| Hellenistic Pherae | V. Chadjitheodorou | 2^nd^-1^st^ century BCE | Cattle | LM3.2 | PH1.9 | -12,10 | -3,56 | - | - | 20,1 |
| Hellenistic Pherae | V. Chadjitheodorou | 2^nd^-1^st^ century BCE | Cattle | LM3.2 | PH1.10 | -11,80 | -3,05 | 0,708329 | 0,000019 | 22,7 |
| Hellenistic Pherae | V. Chadjitheodorou | 2^nd^-1^st^ century BCE | Cattle | LM3.1 | PH2.1 | -9,50 | -2,05 | 0,709490 | 0,000019 | 2,1 |
| Hellenistic Pherae | V. Chadjitheodorou | 2^nd^-1^st^ century BCE | Cattle | LM3.1 | PH2.2 | -9,01 | -1,87 | - | - | 4,5 |
| Hellenistic Pherae | V. Chadjitheodorou | 2^nd^-1^st^ century BCE | Cattle | LM3.1 | PH2.3 | -8,52 | -1,15 | 0,709448 | 0,000018 | 6,6 |
| Hellenistic Pherae | V. Chadjitheodorou | 2^nd^-1^st^ century BCE | Cattle | LM3.1 | PH2.4 | -8,94 | -1,19 | - | - | 8,5 |
| Hellenistic Pherae | V. Chadjitheodorou | 2^nd^-1^st^ century BCE | Cattle | LM3.1 | PH2.5 | -8,99 | -1,04 | 0,709433 | 0,000017 | 11,1 |
| Hellenistic Pherae | V. Chadjitheodorou | 2^nd^-1^st^ century BCE | Cattle | LM3.1 | PH2.6 | -8,25 | -1,46 | - | - | 13,9 |
| Hellenistic Pherae | V. Chadjitheodorou | 2^nd^-1^st^ century BCE | Cattle | LM3.1 | PH2.7 | -8,31 | -1,13 | 0,709424 | 0,000020 | 16,8 |
| Hellenistic Pherae | V. Chadjitheodorou | 2^nd^-1^st^ century BCE | Cattle | LM3.1 | PH2.8 | -9,23 | -2,10 | - | - | 19,7 |
| Hellenistic Pherae | V. Chadjitheodorou | 2^nd^-1^st^ century BCE | Cattle | LM3.1 | PH2.9 | -9,09 | -2,88 | 0,709486 | 0,000017 | 22,6 |
| Hellenistic Pherae | E. Tsoumbekou | 2^nd^-1^st^ century BCE | Sheep | LM3.2 | PH3.1 | -10,88 | -1,61 | 0,709061 | 0,000017 | 3,0 |
| Hellenistic Pherae | E. Tsoumbekou | 2^nd^-1^st^ century BCE | Sheep | LM3.2 | PH3.2 | -12,45 | -3,22 | - | - | 5,5 |
| Hellenistic Pherae | E. Tsoumbekou | 2^nd^-1^st^ century BCE | Sheep | LM3.2 | PH3.3 | -13,10 | -5,24 | 0,709056 | 0,000017 | 7,8 |
| Hellenistic Pherae | E. Tsoumbekou | 2^nd^-1^st^ century BCE | Sheep | LM3.2 | PH3.4 | -12,69 | -5,14 | - | - | 9,9 |
| Hellenistic Pherae | E. Tsoumbekou | 2^nd^-1^st^ century BCE | Sheep | LM3.2 | PH3.5 | -11,36 | -4,72 | 0,709074 | 0,000017 | 12,2 |
| Hellenistic Pherae | E. Tsoumbekou | 2^nd^-1^st^ century BCE | Sheep | LM3.2 | PH3.6 | -11,39 | -4,43 | - | - | 14,6 |
| Hellenistic Pherae | E. Tsoumbekou | 2^nd^-1^st^ century BCE | Sheep | LM3.2 | PH3.7 | -10,36 | -3,04 | - | - | 16,8 |
| Hellenistic Pherae | E. Tsoumbekou | 2^nd^-1^st^ century BCE | Sheep | LM3.2 | PH3.8 | -9,63 | -2,33 | - | - | 18,5 |
| Hellenistic Pherae | E. Tsoumbekou | 2^nd^-1^st^ century BCE | Sheep | LM3.2 | PH3.9 | -9,50 | -1,78 | 0,709211 | 0,000017 | 21,0 |
| Hellenistic Pherae | E. Tsoumbekou | 2^nd^-1^st^ century BCE | Sheep | LM3.2 | PH3.10 | -8,99 | -1,37 | - | - | 24,3 |
| Hellenistic Pherae | E. Tsoumbekou | 2^nd^-1^st^ century BCE | Sheep | LM3.2 | PH3.11 | -9,48 | -1,59 | - | - | 26,4 |
| Hellenistic Pherae | E. Tsoumbekou | 2^nd^-1^st^ century BCE | Sheep | LM3.2 | PH3.12 | -10,39 | -1,43 | 0,709338 | 0,000019 | 28,4 |
| Hellenistic Pherae | V. Chadjitheodorou | 2^nd^-1^st^ century BCE | Sheep | LM3.2 | PH4.1 | -7,75 | -2,13 | 0,709315 | 0,000019 | 5,3 |
| Hellenistic Pherae | V. Chadjitheodorou | 2^nd^-1^st^ century BCE | Sheep | LM3.2 | PH4.2 | -9,71 | -2,25 | - | - | 7,0 |
| Hellenistic Pherae | V. Chadjitheodorou | 2^nd^-1^st^ century BCE | Sheep | LM3.2 | PH4.3 | -9,10 | -0,84 | 0,710444 | 0,000017 | 9,0 |
| Hellenistic Pherae | V. Chadjitheodorou | 2^nd^-1^st^ century BCE | Sheep | LM3.2 | PH4.4 | -9,93 | -1,75 | - | - | 10,7 |
| Hellenistic Pherae | V. Chadjitheodorou | 2^nd^-1^st^ century BCE | Sheep | LM3.2 | PH4.5 | -10,48 | -2,88 | - | - | 12,4 |
| Hellenistic Pherae | V. Chadjitheodorou | 2^nd^-1^st^ century BCE | Sheep | LM3.2 | PH4.6 | -11,35 | -3,63 | 0,709771 | 0,000017 | 15,0 |
| Hellenistic Pherae | V. Chadjitheodorou | 2^nd^-1^st^ century BCE | Sheep | LM3.2 | PH4.7 | -12,15 | -4,21 | - | - | 17,7 |
| Hellenistic Pherae | V. Chadjitheodorou | 2^nd^-1^st^ century BCE | Sheep | LM3.2 | PH4.8 | -10,78 | -5,66 | 0,709087 | 0,000017 | 20,0 |
| Hellenistic Pherae | V. Chadjitheodorou | 2^nd^-1^st^ century BCE | Sheep | LM3.2 | PH4.9 | -9,91 | -5,51 | - | - | 22,7 |
| Hellenistic Pherae | V. Chadjitheodorou | 2^nd^-1^st^ century BCE | Sheep | LM3.2 | PH4.10 | -9,88 | -4,74 | 0,709202 | 0,000017 | 25,0 |
| Hellenistic Pherae | V. Chadjitheodorou | 2^nd^-1^st^ century BCE | Sheep | LM3.1 | PH5.1 | -9,32 | -3,11 | 0,709137 | 0,000020 | 3,2 |
| Hellenistic Pherae | V. Chadjitheodorou | 2^nd^-1^st^ century BCE | Sheep | LM3.1 | PH5.2 | -10,55 | -2,64 | - | - | 5,0 |
| Hellenistic Pherae | V. Chadjitheodorou | 2^nd^-1^st^ century BCE | Sheep | LM3.1 | PH5.3 | -11,87 | -3,75 | 0,709190 | 0,000021 | 7,2 |
| Hellenistic Pherae | V, Chadjitheodorou | 2^nd^-1^st^ century BCE | Sheep | LM3.1 | PH5.4 | -12,14 | -4,22 | - | - | 9.4 |
| Hellenistic Pherae | V. Chadjitheodorou | 2^nd^-1^st^ century BCE | Sheep | LM3.1 | PH5.5 | -14,00 | -6,74 | 0,709009 | 0,000021 | 11,5 |
| Hellenistic Pherae | V. Chadjitheodorou | 2^nd^-1^st^ century BCE | Sheep | LM3.1 | PH5.6 | -11,69 | -4,29 | - | - | 13,4 |
| Hellenistic Pherae | V. Chadjitheodorou | 2^nd^-1^st^ century BCE | Sheep | LM3.1 | PH5.7 | -10,68 | -3,74 | - | - | 15,4 |
| Hellenistic Pherae | V. Chadjitheodorou | 2^nd^-1^st^ century BCE | Sheep | LM3.1 | PH5.8 | -10,44 | -3,87 | 0,708868 | 0,000020 | 17,6 |
| Hellenistic Pherae | V. Chadjitheodorou | 2^nd^-1^st^ century BCE | Sheep | LM3.1 | PH5.9 | -9,37 | -2,97 | - | - | 19,8 |
| Hellenistic Pherae | V. Chadjitheodorou | 2^nd^-1^st^ century BCE | Sheep | LM3.1 | PH5.10 | -7,89 | -1,73 | - | - | 22,9 |
| Hellenistic Pherae | V. Chadjitheodorou | 2^nd^-1^st^ century BCE | Sheep | LM3.1 | PH5.11 | -7,86 | -2,40 | 0,709000 | 0,000020 | 25,5 |
| Hellenistic Pherae | V. Chadjitheodorou | 2^nd^-1^st^ century BCE | Sheep | LM3.2 | PH6.1 | -11,25 | -2,26 | 0,708995 | 0,000020 | 5,0 |
| Hellenistic Pherae | V. Chadjitheodorou | 2^nd^-1^st^ century BCE | Sheep | LM3.2 | PH6.2 | -12,32 | -2,59 | - | - | 7,3 |
| Hellenistic Pherae | V. Chadjitheodorou | 2^nd^-1^st^ century BCE | Sheep | LM3.2 | PH6.3 | -13,16 | -3,63 | - | - | 9,1 |
| Hellenistic Pherae | V. Chadjitheodorou | 2^nd^-1^st^ century BCE | Sheep | LM3.2 | PH6.4 | -12,45 | -4,41 | 0,708992 | 0,000020 | 11,2 |
| Hellenistic Pherae | V. Chadjitheodorou | 2^nd^-1^st^ century BCE | Sheep | LM3.2 | PH6.5 | -12,79 | -4,84 | - | - | 12,9 |
| Hellenistic Pherae | V. Chadjitheodorou | 2^nd^-1^st^ century BCE | Sheep | LM3.2 | PH6.6 | -12,85 | -4,10 | - | - | 14,4 |
| Hellenistic Pherae | V. Chadjitheodorou | 2^nd^-1^st^ century BCE | Sheep | LM3.2 | PH6.7 | -12,78 | -4,22 | 0,708977 | 0,000020 | 16,5 |
| Hellenistic Pherae | V. Chadjitheodorou | 2^nd^-1^st^ century BCE | Sheep | LM3.2 | PH6.8 | -12,27 | -3,51 | - | - | 18,4 |
| Hellenistic Pherae | V. Chadjitheodorou | 2^nd^-1^st^ century BCE | Sheep | LM3.2 | PH6.9 | -12,06 | -3,57 | 0,708966 | 0,000020 | 20,2 |
| Hellenistic Pherae | V. Chadjitheodorou | 2^nd^-1^st^ century BCE | Sheep | LM3.2 | PH6.10 | -10,97 | -2,68 | - | - | 22,2 |
| Hellenistic Pherae | V. Chadjitheodorou | 2^nd^-1^st^ century BCE | Sheep | LM3.2 | PH6.11 | -10,38 | -2,45 | - | - | 24,4 |
| Hellenistic Pherae | V. Chadjitheodorou | 2^nd^-1^st^ century BCE | Sheep | LM3.2 | PH6.12 | -9,89 | -2,26 | 0,708974 | 0,000020 | 27,0 |
| Hellenistic Pherae | V. Chadjitheodorou | 2^nd^-1^st^ century BCE | Sheep | LM3.2 | PH7.1 | -11,28 | -4,82 | 0,708947 | 0,000021 | 3,1 |
| Hellenistic Pherae | V. Chadjitheodorou | 2^nd^-1^st^ century BCE | Sheep | LM3.2 | PH7.2 | -10,53 | -4,17 | - | - | 5,9 |
| Hellenistic Pherae | V. Chadjitheodorou | 2^nd^-1^st^ century BCE | Sheep | LM3.2 | PH7.3 | -9,44 | -4,08 | - | - | 8,1 |
| Hellenistic Pherae | V. Chadjitheodorou | 2^nd^-1^st^ century BCE | Sheep | LM3.2 | PH7.4 | -10,04 | -4,50 | 0,708857 | 0,000020 | 10,5 |
| Hellenistic Pherae | V. Chadjitheodorou | 2^nd^-1^st^ century BCE | Sheep | LM3.2 | PH7.5 | -9,59 | -4,07 | - | - | 12,0 |
| Hellenistic Pherae | V. Chadjitheodorou | 2^nd^-1^st^ century BCE | Sheep | LM3.2 | PH7.6 | -10,58 | -4,72 | - | - | 14,0 |
| Hellenistic Pherae | V. Chadjitheodorou | 2^nd^-1^st^ century BCE | Sheep | LM3.2 | PH7.7 | -11,80 | -4,81 | 0,708826 | 0,000021 | 15,8 |
| Hellenistic Pherae | V. Chadjitheodorou | 2^nd^-1^st^ century BCE | Sheep | LM3.2 | PH7.8 | -11,84 | -4,94 | - | - | 17,9 |
| Hellenistic Pherae | V. Chadjitheodorou | 2^nd^-1^st^ century BCE | Sheep | LM3.2 | PH7.9 | -12,00 | -6,60 | 0,708851 | 0,000021 | 20,3 |
| Hellenistic Pherae | V. Chadjitheodorou | 2^nd^-1^st^ century BCE | Sheep | LM3.2 | PH7.10 | -11,28 | -6,26 | - | - | 22,5 |
| Hellenistic Pherae | V. Chadjitheodorou | 2^nd^-1^st^ century BCE | Sheep | LM3.2 | PH7.11 | -11,05 | -6,05 | - | - | 24,2 |
| Hellenistic Pherae | V. Chadjitheodorou | 2^nd^-1^st^ century BCE | Sheep | LM3.2 | PH7.12 | -9,56 | -5,50 | 0,708952 | 0,000021 | 26,2 |
| Hellenistic Pherae | E. Tsoumbekou | 2^nd^-1^st^ century BCE | Sheep | LM3.1 | PH8.1 | -10,16 | -3,12 | 0,709267 | 0,000020 | 2,2 |
| Hellenistic Pherae | E. Tsoumbekou | 2^nd^-1^st^ century BCE | Sheep | LM3.1 | PH8.2 | -10,95 | -3,53 | - | - | 5,1 |
| Hellenistic Pherae | E. Tsoumbekou | 2^nd^-1^st^ century BCE | Sheep | LM3.1 | PH8.3 | -11,14 | -6,52 | 0,709362 | 0,000021 | 7,9 |
| Hellenistic Pherae | E. Tsoumbekou | 2^nd^-1^st^ century BCE | Sheep | LM3.1 | PH8.4 | -11,09 | -6,12 | - | - | 10,3 |
| Hellenistic Pherae | E. Tsoumbekou | 2^nd^-1^st^ century BCE | Sheep | LM3.1 | PH8.5 | -10,72 | -4,57 | - | - | 12,1 |
| Hellenistic Pherae | E. Tsoumbekou | 2^nd^-1^st^ century BCE | Sheep | LM3.1 | PH8.6 | -10,20 | -4,53 | 0,709167 | 0,000021 | 13,8 |
| Hellenistic Pherae | E. Tsoumbekou | 2^nd^-1^st^ century BCE | Sheep | LM3.1 | PH8.7 | -9,42 | -3,71 | - | - | 15,4 |
| Hellenistic Pherae | E. Tsoumbekou | 2^nd^-1^st^ century BCE | Sheep | LM3.1 | PH8.8 | -9,85 | -2,54 | - | - | 18,0 |
| Hellenistic Pherae | E. Tsoumbekou | 2^nd^-1^st^ century BCE | Sheep | LM3.1 | PH8.9 | -10,50 | -2,37 | 0,709077 | 0,000020 | 20,1 |
| Hellenistic Pherae | E. Tsoumbekou | 2^nd^-1^st^ century BCE | Sheep | LM3.1 | PH8.10 | -11,21 | -4,14 | - | - | 22,1 |
| Hellenistic Pherae | E. Tsoumbekou | 2^nd^-1^st^ century BCE | Sheep | LM3.1 | PH8.11 | -11,24 | -2,15 | - | - | 24,8 |
| Hellenistic Pherae | E. Tsoumbekou | 2^nd^-1^st^ century BCE | Sheep | LM3.1 | PH8.12 | -11,12 | -2,89 | 0,709128 | 0,000020 | 26,8 |
| Hellenistic Pherae | V. Chadjitheodorou | 2^nd^-1^st^ century BCE | Goat | LM3.2 | PH9.1 | -11,50 | -3,62 | 0,709533 | 0,000020 | 3,4 |
| Hellenistic Pherae | V. Chadjitheodorou | 2^nd^-1^st^ century BCE | Goat | LM3.2 | PH9.2 | -10,81 | -2,15 | - | - | 6,6 |
| Hellenistic Pherae | V. Chadjitheodorou | 2^nd^-1^st^ century BCE | Goat | LM3.2 | PH9.3 | -10,67 | -1,63 | 0,709499 | 0,000020 | 9,5 |
| Hellenistic Pherae | V. Chadjitheodorou | 2^nd^-1^st^ century BCE | Goat | LM3.2 | PH9.4 | -11,30 | -1,41 | - | - | 11,6 |
| Hellenistic Pherae | V. Chadjitheodorou | 2^nd^-1^st^ century BCE | Goat | LM3.2 | PH9.5 | -11,76 | -3,98 | 0,709347 | 0,000021 | 13,7 |
| Hellenistic Pherae | V. Chadjitheodorou | 2^nd^-1^st^ century BCE | Goat | LM3.2 | PH9.6 | -12,58 | -4,31 | - | - | 16,1 |
| Hellenistic Pherae | V. Chadjitheodorou | 2^nd^-1^st^ century BCE | Goat | LM3.2 | PH9.7 | -12,88 | -5,65 | 0,709375 | 0,000020 | 17,9 |
| Hellenistic Pherae | V. Chadjitheodorou | 2^nd^-1^st^ century BCE | Goat | LM3.2 | PH9.8 | -10,57 | -4,12 | 0,709438 | 0,000020 | 21,0 |
| Hellenistic Pherae | V. Chadjitheodorou | 2^nd^-1^st^ century BCE | Sheep | LM3.1 | PH10.1 | -10,38 | -3,08 | 0,708881 | 0,000021 | 2,1 |
| Hellenistic Pherae | V. Chadjitheodorou | 2^nd^-1^st^ century BCE | Sheep | LM3.1 | PH10.2 | -10,45 | -2,41 | - | - | 4,4 |
| Hellenistic Pherae | V. Chadjitheodorou | 2^nd^-1^st^ century BCE | Sheep | LM3.1 | PH10.3 | -10,18 | -3,06 | - | - | 6,7 |
| Hellenistic Pherae | V. Chadjitheodorou | 2^nd^-1^st^ century BCE | Sheep | LM3.1 | PH10.4 | -10,24 | -4,36 | 0,709983 | 0,000020 | 9,0 |
| Hellenistic Pherae | V. Chadjitheodorou | 2^nd^-1^st^ century BCE | Sheep | LM3.1 | PH10.5 | -10,74 | -5,66 | - | - | 10,8 |
| Hellenistic Pherae | V. Chadjitheodorou | 2^nd^-1^st^ century BCE | Sheep | LM3.1 | PH10.6 | -11,26 | -5,79 | - | - | 12,5 |
| Hellenistic Pherae | V. Chadjitheodorou | 2^nd^-1^st^ century BCE | Sheep | LM3.1 | PH10.7 | -10,31 | -6,60 | - | - | 14,4 |
| Hellenistic Pherae | V. Chadjitheodorou | 2^nd^-1^st^ century BCE | Sheep | LM3.1 | PH10.8 | -11,03 | -4,66 | 0,709729 | 0,000021 | 17,0 |
| Hellenistic Pherae | V. Chadjitheodorou | 2^nd^-1^st^ century BCE | Sheep | LM3.1 | PH10.9 | -10,39 | -4,59 | - | - | 19,3 |
| Hellenistic Pherae | V. Chadjitheodorou | 2^nd^-1^st^ century BCE | Sheep | LM3.1 | PH10.10 | -10,28 | -4,30 | 0,708823 | 0,000020 | 21,7 |
| Hellenistic Pherae | V. Chadjitheodorou | 2^nd^-1^st^ century BCE | Sheep | LM3.1 | PH10.11 | -10,58 | -4,29 | - | - | 23,8 |
| Hellenistic Pherae | V. Chadjitheodorou | 2^nd^-1^st^ century BCE | Sheep | LM3.1 | PH10.12 | -10,76 | -3,48 | 0,708648 | 0,000020 | 26,7 |
| Hellenistic Pherae | N. Tsekou | Late 3^rd^-1^st^ century BCE | Sheep | LM3.2 | PH11.1 | -6,86 | -2,90 | 0,708708 | 0,000020 | 5,9 |
| Hellenistic Pherae | N. Tsekou | Late 3^rd^-1^st^ century BCE | Sheep | LM3.2 | PH11.2 | -6,84 | -2,55 | - | - | 8,7 |
| Hellenistic Pherae | N. Tsekou | Late 3^rd^-1^st^ century BCE | Sheep | LM3.2 | PH11.3 | -8,01 | -1,63 | 0,708712 | 0,000017 | 11,1 |
| Hellenistic Pherae | N. Tsekou | Late 3^rd^-1^st^ century BCE | Sheep | LM3.2 | PH11.4 | -10,54 | -2,56 | - | - | 13,6 |
| Hellenistic Pherae | N. Tsekou | Late 3^rd^-1^st^ century BCE | Sheep | LM3.2 | PH11.5 | -11,37 | -3,08 | - | - | 15,6 |
| Hellenistic Pherae | N. Tsekou | Late 3^rd^-1^st^ century BCE | Sheep | LM3.2 | PH11.6 | -12,15 | -4,36 | 0,708553 | 0,000017 | 17,6 |
| Hellenistic Pherae | N. Tsekou | Late 3^rd^-1^st^ century BCE | Sheep | LM3.2 | PH11.7 | -12,16 | -4,71 | - | - | 19,8 |
| Hellenistic Pherae | N. Tsekou | Late 3^rd^-1^st^ century BCE | Sheep | LM3.2 | PH11.8 | -11,80 | -5,76 | 0,708673 | 0,000017 | 22,3 |
| Hellenistic Pherae | N. Tsekou | Late 3^rd^-1^st^ century BCE | Sheep | LM3.2 | PH11.9 | -10,50 | -4,80 | - | - | 25,2 |
| Hellenistic Pherae | N. Tsekou | Late 3^rd^-1^st^ century BCE | Sheep | LM3.2 | PH11.10 | -8,79 | -3,82 | - | - | 27,9 |
| Hellenistic Pherae | N. Tsekou | Late 3^rd^-1^st^ century BCE | Sheep | LM3.2 | PH11.11 | -8,43 | -4,83 | 0,708977 | 0,000017 | 30,6 |

**S7 Table. Isotopic data (δ^13^C, δ^18^O, and ^87^Sr/ ^86^Sr) recorded from caprine and cattle mandibular third and second molar enamel according to distance from the enamel-root.**
