# Supplementary material for: Where did the herds go? Combining zooarchaeological and isotopic data to examine animal management in ancient Thessaly (Greece)
Source: PLoS One. 2024 Oct 22;19(10):e0299788. doi: 10.1371/journal.pone.0299788 (PMC11495569; doi:10.1371/journal.pone.0299788)
Supplement: S8 Table — (DOCX) [file pone.0299788.s014.docx]

Supporting Information- Tables

| **Sample ID** | **Type** | **Altitude** | **Location** | | | **Geological formations** | **Period** | **^87^Sr/^86^Sr** | **2σ** |
| --- | --- | --- | --- | --- | --- | --- | --- | --- | --- |
|  |  |  | **Latitude** | **Longtitude** | |  |  |  |  |
| QP.1.2 | *Quercus pubescens* | 1089m | Othrys Mountain | | | Upper Cretaceous flysch. Shales, sand-stones, conglomerates and intercalated limestones. Fossils. | Upper Cretaceous | 0.709071 | 0,000054 |
|  |  |  | +39.049 | +22.740 | |  |  |  |  |
| QP.1.3 | *Quercus pubescens* | 1112m | Othrys Mountain | | | Upper Cretaceous flysch. Shales, sand-stones, conglomerates and intercalated limestones. Fossils. | Upper Cretaceous | 0.709426 | 0,000028 |
|  |  |  | +39.050 | +22.739 | |  |  |  |  |
| QP.2.2 | *Quercus pubescens* | 965m | Othrys Mountain | | | Upper Cretaceous transgressif limestones. Platy limestones with Radiolites. Massive limestones with Hipurites, Orbitoides etc. | Upper Cretaceous | 0.708212 | 0,000053 |
|  |  |  | +39.018 | +22.780 | |  |  |  |  |
| QP.2.3 | *Quercus pubescens* | 970m | Othrys Mountain | | | Upper Cretaceous transgressif limestones. Platy limestones with Radiolites. Massive limestones with Hipurites, Orbitoides etc. | Upper Cretaceous | 0.708122 | 0,000054 |
|  |  |  | +39.018 | +22.780 | |  |  |  |  |
| HA2 | *Ovis aries* bone | - | Magoula Plataniotiki | | | Quaternary undivided, diluvium and alluvium. Clays, sands, gravels, talus.  Coastal conglomerates. Continental deposits. | Quaternary | 0.708857 | 0,000012 |
|  |  |  | +39.163 | | +22.843 |  |  |  |  |
| HA10 | *Bos taurus* bone | - | Magoula Plataniotiki | | | Quaternary undivided, diluvium and alluvium. Clays, sands, gravels, talus.  Coastal conglomerates. Continental deposits. | Quaternary | 0.708830 | 0,000012 |
|  |  |  | +39.163 | | +22.841 |  |  |  |  |
| NH4 | *Ovis aries* bone | - | New Halos | | | Quaternary undivided, diluvium and alluvium. Clays, sands, gravels, talus.  Coastal conglomerates. Continental deposits. | Quaternary | 0.708690 | 0,000017 |
|  |  |  | +39.147 | | +22.826 |  |  |  |  |
| PH3 | *Ovis aries* bone | - | Pherae | | | Alluvial deposits light-grey to brown-grey fluvio-lacustrine material of silt, clay, and very little coarser material deposited in the Voiviis (Karla) lake basin, deposits on plains, open towards the sea, and small interior basins of clay, sand, and pebbles, torrential deposits, torrential terraces material, and eluvial mantle material. | Quaternary/ Holocene | 0.709086 | 0,000017 |
|  |  |  | +39.381 | | +22.740 |  |  |  |  |
| PH11 | *Ovis aries* dentine | - | Pherae | | |  | Quaternary/ Holocene | 0.708976 | 0,000017 |
|  |  |  | +39.383 | | +22.741 |  |  |  |  |

**S8 Table. Strontium isotopic ratios (^87^Sr/^86^Sr) obtained on modern tree leaves and animal bones from different geologic formations.**
